# Chromosomal Speciation in the Genomics Era: Disentangling Phylogenetic Evolution of Rock-wallabies

**DOI:** 10.3389/fgene.2017.00010

**Published:** 2017-02-10

**Authors:** Sally Potter, Jason G. Bragg, Mozes P. K. Blom, Janine E. Deakin, Mark Kirkpatrick, Mark D. B. Eldridge, Craig Moritz

**Affiliations:** ^1^Research School of Biology, Australian National University, ActonACT, Australia; ^2^Australian Museum Research Institute, Australian Museum, SydneyNSW, Australia; ^3^National Herbarium of New South Wales, The Royal Botanic Gardens and Domain Trust, SydneyNSW, Australia; ^4^Department of Bioinformatics and Genetics, Swedish Museum of Natural HistoryStockholm, Sweden; ^5^Institute for Applied Ecology, University of Canberra, BruceACT, Australia; ^6^Department of Integrative Biology, University of Texas, AustinTX, USA

**Keywords:** chromosome rearrangement, speciation, rock-wallaby, divergence, genomics

## Abstract

The association of chromosome rearrangements (CRs) with speciation is well established, and there is a long history of theory and evidence relating to “chromosomal speciation.” Genomic sequencing has the potential to provide new insights into how reorganization of genome structure promotes divergence, and in model systems has demonstrated reduced gene flow in rearranged segments. However, there are limits to what we can understand from a small number of model systems, which each only tell us about one episode of chromosomal speciation. Progressing from patterns of association between chromosome (and genic) change, to understanding processes of speciation requires both comparative studies across diverse systems and integration of genome-scale sequence comparisons with other lines of evidence. Here, we showcase a promising example of chromosomal speciation in a non-model organism, the endemic Australian marsupial genus *Petrogale*. We present initial phylogenetic results from exon-capture that resolve a history of divergence associated with extensive and repeated CRs. Yet it remains challenging to disentangle gene tree heterogeneity caused by recent divergence and gene flow in this and other such recent radiations. We outline a way forward for better integration of comparative genomic sequence data with evidence from molecular cytogenetics, and analyses of shifts in the recombination landscape and potential disruption of meiotic segregation and epigenetic programming. In all likelihood, CRs impact multiple cellular processes and these effects need to be considered together, along with effects of genic divergence. Understanding the effects of CRs together with genic divergence will require development of more integrative theory and inference methods. Together, new data and analysis tools will combine to shed light on long standing questions of how chromosome and genic divergence promote speciation.

## Introduction

Differences in how the genome is packaged – chromosome variation – have long been known to influence how genetic variation is transmitted and redistributed within and among populations (Darlington, 1958; White, 1973). Today, with increasing availability of high quality genome assemblies, the capacity for genome-scale resequencing and the tools of molecular cytogenetics and population- and phylo-genomic analysis, we are returning to a whole-genome perspective on evolution. At the same time, evidence from genome comparisons is revealing that reticulate evolution, including introgression across distantly related species, is far more common in animals than previously thought (Mallet et al., 2016) with implications for gene-tree – species-tree discordance (Edwards et al., 2016). This directs attention to the potential for differing extents of introgression within and outside rearranged regions of the genome (Noor and Bennett, 2009; Crawford et al., 2015).

Here, we revisit the early history of thinking about how chromosomal rearrangements (CRs) affect population and speciation processes. We then highlight a case study that emphasizes how a combined knowledge of genome architecture and genomic sequence divergence is important for understanding the history of CRs in association with speciation and being able to assess whether gene flow is reduced in rearranged regions. Finally, we return to broader themes, considering what combinations of evidence and theory are necessary to gain a holistic understanding of how chromosome change can promote incipient divergence and ultimately translate into species diversification.

### Chromosome Change, Population Processes, and Speciation – a Potted History

Observations on differences in chromosome number and form were some of the earliest data available on genetic differences among species. Inevitably, this led to consideration of whether and how such large-scale restructuring of the genome could cause reproductive isolation – speciation – as well as the role of CRs in adaptive evolution within species (Sturtevant, 1938; Dobzhansky, 1950; Stebbins, 1950; Grant, 1964; White, 1973).

The initial focus on adaptive evolution of CRs was largely for paracentric inversions, and their role in recombination suppression and thus accumulation of linked adaptive genes (Dobzhansky, 1950). More broadly, consideration of how multiple CRs (e.g., reciprocal translocations) could lead to long chains of chromosomes with no recombination lead to concepts of “genetic systems” and their role in maintaining heterozygosity (Darlington, 1958; James, 1982). This thread connecting chromosome organization with adaptive evolution continues today with the proposal that the recombination suppression associated with CRs can promote local adaptation and the accumulation of genetic incompatibilities between species (Navarro and Barton, 2003; Kirkpatrick and Barton, 2006; reviewed in Faria and Navarro, 2010; Ortiz-Barrientos et al., 2016). In one powerful example, Shaw et al. (1986) found that shifts in recombination positions in chromosome heterozygotes of *Caledia* grasshoppers was associated with hybrid breakdown, and more so than genetic distance *per se*. Association between range size and rate of inversions in birds also support these models, albeit indirectly (Hooper and Price, 2015).

As evidence of marked differences in chromosome organization among species continued to accumulate, various concepts of chromosomal speciation developed (reviewed by White, 1978; King, 1993). For the most part, these focused on types of CRs that potentially reduce fertility of heterozygotes – “sterility models” – because of disruptions of segregation, or meiotic silencing of unsynapsed chromosomes (MSUC) during meiosis (Garagna et al., 2014). The obvious challenge is to explain how a new mutation that reduces the fitness of its heterozygous carrier can survive selection against it, to establish within a local population. Stimulated by the observation that such changes are often seen in taxa that form small isolated populations (e.g., Bush et al., 1977), various models based on strong genetic drift or founder events followed, some analogous to Wright’s Shifting Balance Theory of alternating drift and adaptive evolution in metapopulations (Wright, 1982). Such models were immediately controversial, especially when they invoked variants of sympatric speciation (Key, 1968; Futuyma and Mayer, 1980) or rapid fixation in founder populations (Templeton, 1981). This led to strong skepticism of the view that individual chromosome changes, though reduced fertility, could be a primary and common driver of speciation (Walsh, 1982; Coyne et al., 2000; Coyne and Orr, 2004). Nonetheless, in chromosomally diverse butterflies and *Drosophila*, differences in chromosome number accumulate more rapidly between sympatric than allopatric species and are linked to reinforcing selection for pre-mating isolation (Noor et al., 2001; Lukhtanov et al., 2005; Kandul et al., 2007). An association between speciation and chromosomal evolution was identified in mammals (Bush et al., 1977), and more recently in a diverse genus of lizards, *Sceloporus*, where a phylogenomic analysis revealed higher speciation rates in clades with extensive Robertsonian fusions (Leaché et al., 2016).

Mechanisms that could promote fixation of chromosome changes despite reduced hybrid fertility include: (i) meiotic drive, (ii) establishment of recombination suppression which facilitates adaptive evolution, and simply, (iii) beneficial effects of CRs on gene expression. (i) *Meiotic drive* – (segregation distortion) is a powerful evolutionary force that can drive mutations that otherwise reduce fitness to fixation by biased transmission of chromosomes (reviewed in Lindholm et al., 2016; see also Pardo-Manuel de Villena and Sapienza, 2001). Meiotic drive has been observed to favor Robertsonian fusions (metacentric) over unfused (acrocentric) chromosomes in shrews (Wyttenbach et al., 1998; Fedyk and Chetnicki, 2007) but evidence for this in *Mus* is mixed (Nachman and Searle, 1995; Chmátal et al., 2014). Meiotic drive might also underpin large-scale patterns of chromosome diversity in fish (Yoshida and Kitano, 2012; Molina et al., 2014). Sex chromosomes have been shown to be frequently involved in fusions in fish and amniotes (see Pokorná et al., 2014; Pennell et al., 2015). (ii) *Recombination suppression and adaptation* – selection to reduce negative effects of chromosomal heterozygosity, including shifts in recombination (chiasma) positions, non-homologous pairing and synaptic adjustment. For synapsis to occur during meiosis, chromosomes need to pair to allow crossing over and this process uses the synaptonemal complex. Evidence from a variety of organisms – mice (Johannisson and Winking, 1994; Borodin et al., 2005; Manterola et al., 2009), humans (Guichaoua et al., 1986), chickens (Kaelbling and Fechheimer, 1985) and *Caenorhabditis elegans* (Henzel et al., 2011), highlight that homology of chromosomes is not required to complete this process and synaptic adjustment (reviewed in Zickler and Kleckner, 1999) can overcome issues of non-homology. There is also evidence that this occurs broadly in eutherian mammals between the sex chromosomes (pairing of X and Y), where only a short domain is homologous (pseudo-autosomal region) allowing for non-homologous synapsis (Bergero and Charlesworth, 2009). However, the ability to overcome non-homology depends on a number of factors including the size of the rearrangement, the gene content, the location with respect to centromeres and telomeres and the genetic background (see Torgasheva and Borodin, 2010). This can favor production of balanced gametes for a variety of rearrangements including deletions, insertions, inversions, Robertsonian fusions (Kingswood et al., 1994; Vozdova et al., 2014) and duplications (reviewed in Torgasheva and Borodin, 2010). In addition, recombination suppression may drive adaptive evolution by bringing together advantageous gene combinations (Hoffmann and Rieseberg, 2008; see also Navarro and Barton, 2003). Theory on effects of recombination suppression focuses primarily on inversions (e.g., Kirkpatrick and Barton, 2006) but also considers fusions (Guerrero and Kirkpatrick, 2014) and centric shifts, which may occur via pericentric inversion, three break rearrangements or establishment of neocentromeres, and in the vicinity of centromeres involved in fusion/fissions events (Rieseberg, 2001; Navarro and Barton, 2003). Finally, the simplest possibility is (iii) *a beneficial mutation* – a rearrangement could generate a beneficial effect of relocating genes into a different regulatory environment, long referred to as position effects (Muller, 1930). As with most mutations, such changes will most often be deleterious (as in humans – Harewood and Fraser, 2014)

The well-known Bateson Dobzhansky Muller (BDM) model (based on work of Bateson, 1909; Dobzhansky, 1936; Muller, 1942) can operate for CRs as it does for genic mutations, avoiding the hybrid-sterility conundrum. Independent chromosome changes arise within isolates, and proceed to fixation by drift or adaptive evolution, followed, on secondary contact, by reduced fertility of heterozygotes for multiple rearrangements (see Coyne and Orr, 2004). Comparative and experimental data on *Mus* (reviewed in Garagna et al., 2014), *Sorex* shrews (Polyakov et al., 2011; Horn et al., 2012) and *Rhogeessa* bats (Baird et al., 2009), appear to be exemplify the BDM process, where the focus is on systems with multiple chromosomal fusions with one or more common arms in different fusion arrangements, i.e., monobrachial homology (Baker and Bickham, 1986).

Putting aside contention over whether chromosomal speciation is common, empirical systems where closely related species differ by multiple, complex CRs are frequently observed (White, 1973; King, 1993; Coyne and Orr, 2004; Dobigny et al., 2017). However, our current understanding of CRs is largely based on changes that are visible by classical cytology and chromosome banding. With the tools of molecular cytogenetics and high resolution genome sequencing, yet more, often substantial, CRs are being discovered between species thought to have few changes (e.g., human vs. chimpanzee; Prado-Martinez et al., 2013; Farré et al., 2015).

So, how do we revisit these old questions and debates with new theory and empirical evidence? Despite recent advances in chromosomal speciation theory (Kirkpatrick and Barton, 2006; Faria and Navarro, 2010; Kirkpatrick, 2010, 2017; Guerrero et al., 2012b; Guerrero and Kirkpatrick, 2014), more needs to be done to develop inference methods that can exploit genomic comparisons (see Prospectus section). From the empirical perspective, one fruitful approach is to apply genome-scale analyses to systems that exemplify chromosome change among closely related taxa. Sites and Moritz (1987) proposed that models of chromosomal speciation that require strong genetic drift could be tested using simple predictions for reduced genetic polymorphism and elevated divergence, but both the empirical and inference tools available at the time were limiting. This has now changed substantially, with the ability to sequence thousands of loci across populations of any organism and to use coalescent and network methods to infer divergence history (Edwards et al., 2016). The key challenge for recently diverged taxa is to disentangle the effects of retained ancestral polymorphism (incomplete lineage sorting – ILS) from subsequent gene flow. While this remains challenging, the emergence of isolation-with-migration models (Pinho and Hey, 2010) and phylogenetic network methods (Nakhleh, 2013), when combined with genome-scale data, offer some hope. Recent research into the *Anopheles* system has highlighted the value of genomic data in disentangling ILS from introgression (Fontaine et al., 2015; Wen et al., 2016), as has sliding window analysis of genomes in *Xiphophorus* fishes (Cui et al., 2013). Whole genomes allow for a suite of new analyses to identify introgression (e.g., using the ABBA-BABA discordance test; Green et al., 2010; Durand et al., 2011; Martin et al., 2015; Nater et al., 2015), but currently there are still limitations based on genome sequencing and alignments, where phasing errors can lead to over-estimation of recombination or mutations (e.g., Qi et al., 2014; Rasmussen et al., 2014). Further, it may be that comparative genome screening alone will not be sufficient to resolve different effects of CRs on divergence (e.g., Suh, 2016; but see Prospectus).

### Inferring Divergence Histories of Candidates for Chromosomal Speciation

To resolve whether CRs initiate divergence or follow genic speciation, we need to focus on recently diverged taxa (Coyne and Orr, 2004). We need to identify organisms that can help address questions in chromosomal speciation and apply integrative tools to them. In particular, cytogenetic and molecular data can be combined to infer the sequence and timing of CRs in systems with complex chromosome change (Faria and Navarro, 2010). This is especially important to interpret signatures of genetic divergence associated with these CRs (Noor and Bennett, 2009). It should be reiterated that it remains a formidable challenge to resolve relationships and reticulations among recently separated species (e.g., Leaché et al., 2016).

Several recent comparative studies of species with high quality reference genomes have used extensive resequencing to resolve divergence histories and contrast levels of introgression among recently separated taxa that differ by chromosomal inversions (e.g., Primates – Carbone et al., 2014, *Drosophila* – Kulathinal et al., 2009; McGaugh and Noor, 2012; Lohse et al., 2015; *Anopheles* – Wen et al., 2016). There has, however, been mixed support for recombination suppression models (see Faria and Navarro, 2010). By contrast to chromosomal inversions, there have been few genome-scale analyses of closely related taxa with complex Robertsonian fusions. In the Robertsonian fusion races of *Mus*, increased genetic divergence has been observed at microsatellite loci near the centromeres of fused chromosomes (Franchini et al., 2010; Förster et al., 2016) and simulations of recombination suppression versus hybrid breakdown reveal that hybrid breakdown alone could explain the patterns in *Mus* from Italy (Giménez et al., 2013). Like *Mus*, reduced gene flow (higher divergence) is evident within CRs in *Sorex* shrews (Basset et al., 2006; Yannic et al., 2009).

While analyses of model systems, such as the above, have provided important insights into causes and consequences of CRs, it remains important to extend analyses of effects of chromosome change to systems with distinct genomic features and population structures (Payseur and Rieseberg, 2016). In the following, we present one such example and then conclude with a prospectus for how to advance this and other non-model systems. With this and other such systems, we hope to obtain a greater insight into the processes driving variation in genomic architecture, that lead to divergence and speciation.

## Case Study: *Petrogale* Rock-Wallabies

**FIGURE 1:**
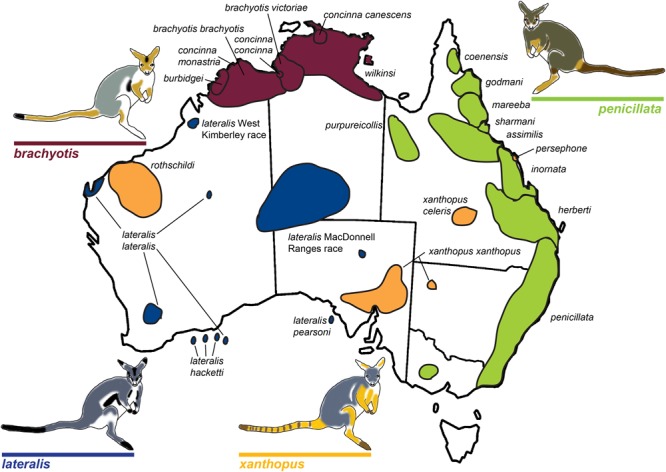
**Map of rock-wallaby (*Petrogale*) taxa distributions across Australia.** Map modified from Eldridge and Close (1993). Taxa are colored in accordance with their chromosomal groupings: the *brachyotis* group = red; the *xanthopus* group = yellow; the *lateralis* group = blue; and the *penicillata* group = green.

The rock-wallaby (*Petrogale*) system has been considered a classical model for chromosomal speciation due to extensive chromosome repatterning, combined with their habitat specialization (rocky environments) which causes populations to be isolated and small (King, 1993). Rock-wallabies are medium sized marsupials (1–12 kg) that inhabit complex rocky areas distributed across continental Australia and some offshore islands, (Eldridge, 2008). A strong propensity for isolation among disjunct rocky habitats (e.g., Pope et al., 1996; Hazlitt et al., 2006) is thought to increase their rate of speciation and contribute to the fixation of novel CRs (Eldridge and Close, 1993). *Petrogale* includes 17 recognized species corresponding to 23 chromosomal taxa (Supplementary Table 1; **Figure 1**). This is the most chromosomally diverse genus of marsupials, which in general have a conserved karyotype across all five Australasian and American super-families (2*n* = 14; Rofe and Hayman, 1985; Hayman, 1990; see O’Neill et al., 1999; Graves and Renfree, 2013). Macropodids (kangaroos and wallabies) show variable karyotypes (see O’Neill et al., 1999), but the ancestral macropodid 2*n* = 22 karyotype is only found in *Petrogale* (*P. lateralis, P. persephone, P. rothschildi*, and *P. xanthopus*; Eldridge et al., 1992a) and *Thylogale* (Pademelons). The 2*n* = 22 macropodid ancestral karyotype is itself derived from the widespread 2*n* = 14 marsupial karyotype by a series of fissions (Rofe, 1979; Hayman, 1990). CRs are extensive across *Petrogale*, and range from simple to complex. A majority of the rearrangements are Robertsonian fusions, but there is also cytogenetic evidence for inversions and centric shifts (centromeric transpositions) (Eldridge et al., 1989, 1990, 1991, 1992b; Eldridge and Close, 1992, 1993). In addition, the X chromosome is frequently variable in morphology among taxa and also sometimes within taxa (Eldridge and Close, 1997). The highest chromosomal diversity occurs in groups that are sympatric or parapatric (*brachyotis* and *penicillata* groups; Eldridge et al., 1992b; Eldridge and Close, 1993; **Figure 1**), and these are also the most speciose. This pattern matches the predictions of chromosomal speciation models (see Faria and Navarro, 2010) and the recombination suppression model. This could reflect yet another scenario where fixation rate of chromosomal rearrangements correlates with parapatry and sympatry – suggesting adaptation and divergent selection could be a dominant process driving fixation (e.g., Hooper and Price, 2015).

The *brachyotis* group of rock-wallabies includes four species and five sub-species distributed across northwestern Australia which have the most complex rearrangements found in *Petrogale* (Supplementary Table 2) and large amounts of centromeric constitutive heterochromatin not present in other *Petrogale* (Maynes, 1989; Sharman et al., 1990; Eldridge et al., 1992b; Eldridge and Close, 1993). Within the *lateralis* group, there are two chromosomal races and three sub-species (Eldridge et al., 1991; **Figure 1**). These races/sub-species are recently diverged and are distinguished by single autosomal rearrangements or fusions (Eldridge and Close, 1993, 1997; **Figure 1**). However, the most interesting group are the recently diverged (∼0.5–2.7 mya; Potter et al., 2012a) Queensland *penicillata* group taxa. Six parapatric species display extensive variation in karyotypes ranging from simple to complex – including fusions, inversions and centric shifts (see Eldridge and Close, 1993). Early research was driven by cytogenetic analyses (reviewed in Eldridge and Close, 1997) and captive breeding experiments that showed evidence of reproductive isolation including infertile male hybrids and reduced fertility of female hybrids (Eldridge and Close, 1992). This resulted in the description of three new species (Eldridge and Close, 1992) and a focus on the role of chromosomal variation in speciation. Meiotic irregularities, including problems with more extensive rearrangements and X-autosome associations have been reported (Close et al., 1996) – patterns also seen in model systems (e.g., *Mus*). Recent genetic analysis of the *penicillata* group using microsatellites and mitochondrial DNA (mtDNA) found extensive sharing of alleles between some of the most chromosomally divergent species (Potter et al., 2015). This could be a consequence of introgression or ILS. Further analysis of nuclear markers across the genome is required to assess the genomic divergence between these species and assess if speciation with gene flow is occurring between these taxa, or if more complex interactions between genomic architecture and genic divergence is at play. The characteristics of this genus, specifically their rapid radiation and extensive chromosome variation, make them a valuable model for understanding chromosome evolution and speciation.

Phylogenetic analyses of the rock-wallabies have not previously included representatives of all 23 chromosomal taxa, nor have they been able to resolve phylogenetic relationships, particularly among the more recently evolved species within the *penicillata* group (Campeau-Péloquin et al., 2001; Potter et al., 2012a). This, in addition to evident homoplasy of rearrangements (see Eldridge and Close, 1993), has precluded tracing the evolution of chromosomal changes. The phylogenetic relationship of *P. xanthopus* and *P. purpureicollis* has also been difficult to resolve (see Eldridge et al., 1991; Eldridge and Close, 1993; Potter et al., 2012a), which has hindered interpreting chromosome evolution as these taxa retain the ancestral chromosome number.

Here, we report results from targeted capture for ∼2000 exons from two individuals per taxon to resolve the relationships across the genus (all supplementary material and methods are outlined in Supplementary Datasheet 1). These data allow us to understand the evolution of chromosomes in this group. In particular, they provide insight into phylogenetic and sequence divergence signals of discordance across the X, rearranged and non-rearranged chromosome arms that could reflect effects of CRs on gene flow (see Supplementary Table 3). We focus on sets of concatenated loci, rather than individual gene trees as individual exons have low phylogenetic resolution at this scale. While it is desirable to use multispecies coalescent approaches (e.g., ^∗^BEAST and ASTRAL), such programs are confounded by introgression across non-sister data and are therefore unsuitable for this system (see Solis-Lemus et al., 2016). Hence, we explore multispecies coalescent network approaches that allow for introgression (see below; reviewed in Nakhleh, 2013; Edwards et al., 2016). We expect to find discordant phylogenies and divergence levels between these categories of loci, particularly for the recent radiation of Queensland taxa.

**FIGURE 2:**
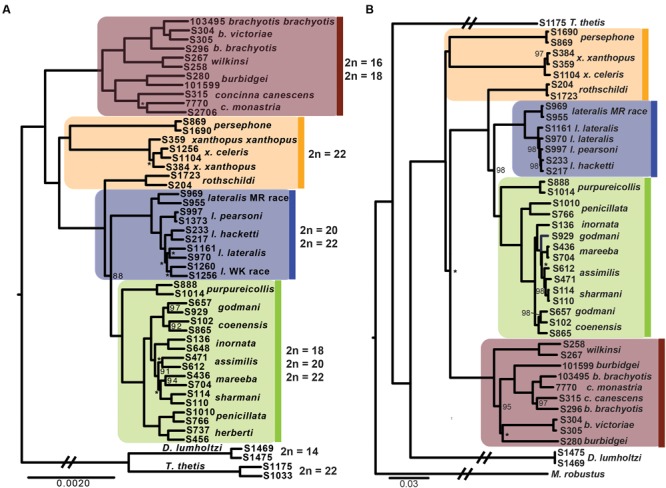
**(A)** Phylogenetic relationships of rock-wallabies (*Petrogale*) based on a maximum likelihood analysis of concatenated nuclear data (1961 loci). Bootstrap support < 100% is outlined on the nodes, ^∗^ = < 50%; nodes with neither have support of 100%. Four chromosomal groups are highlighted on the phylogeny: the *brachyotis* group = red; the *xanthopus* group = yellow; the *lateralis* group = blue; and the *penicillata* group = green. Karyotype variation (2*n*) for each of the four chromosomal groups is highlighted. *Dendrolagus lumholtzi* (tree kangaroo) and *Thylogale thetis* (pademelon) are used as outgroups. **(B)** A maximum likelihood mitochondrial phylogeny of *Petrogale* based on all mitochondrial coding genes (12 loci). Chromosomal groups are highlighted to match **(A)**, as is bootstrap support.

The phylogenetic relationships amongst taxa using the entire dataset of 1961 exons and ∼1 million bp firmly resolves, for the first time, relationships within *Petrogale* (**Figure 2**). The *brachyotis* group with the most extensive chromosomal rearrangements is also phylogenetically basal, which is consistent with previous genetic data (Campeau-Péloquin et al., 2001; Potter et al., 2012a). The *xanthopus* chromosomal group is paraphyletic. *P. rothschildi* forms the sister taxon to the *lateralis* and *penicillata* groups, where as *P. persephone* and *P. xanthopus* form a well supported monophyletic group. There is deep divergence between all three taxa that retain the ancestral karyoptype. The cytogenetically conservative *lateralis* group has similar branch lengths among taxa to those among species within the more chromosomally diverse *penicillata* group. Despite recent speciation, each taxon within the *lateralis* and *penicillata* groups is monophyletic, albeit with lower support for *P. lateralis lateralis, P. l.* West Kimberley race and *P. assimilis*. Relationships amongst some of the most closely related taxa are also not strongly resolved, but we note that monophyly of *P. mareeba* and *P. sharmani* is consistent with their chromosomal evolutionary history, since both share a derived fusion between chromosomes 5 and 10. Although the phylogenetic position of *P. purpureicollis* has been previously unresolved (Sharman et al., 1990; Eldridge et al., 1991; Campeau-Péloquin et al., 2001; Potter et al., 2012a), these new data strongly resolve it as sister to the *penicillata* group. The sub-species of both *P. brachyotis* and *P. xanthopus* (with no known chromosomal differences) did not form monophyletic lineages, suggesting recent divergence or some nuclear gene flow.

**FIGURE 3:**
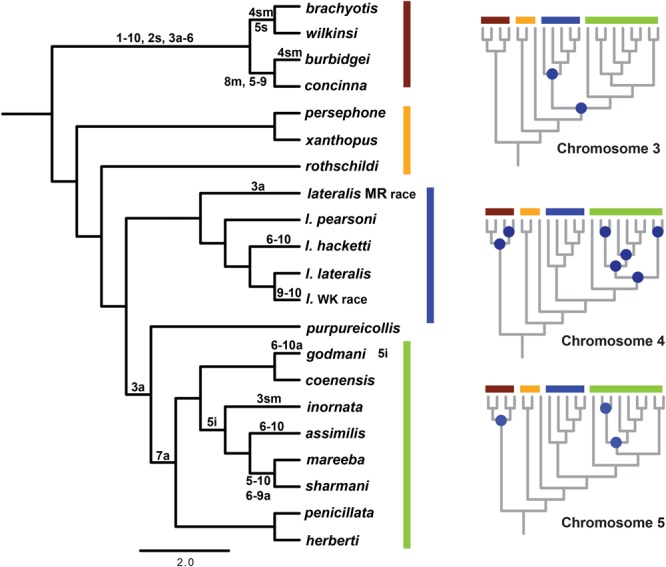
**Reconstruction of chromosomal rearrangements based on parsimony analysis for *Petrogale* using only known chromosomal karyotypes (e.g., no sub-species for *P. brachyotis, P. concinna* or *P. xanthopus*).** Reconstructions of ancestral karyotypes are highlighted on the main phylogeny and those that could not be resolved for nodes in the phylogeny are indicated in blue for chromosomes 3, 4, and 5. See Supplementary Table 2 for character state matrix. Chromosomal groups are again outlined in color: *brachyotis* = red; *xanthopus* = yellow; *lateralis* = blue; and *penicillata* = green. Chromosomal rearrangements include: centric shifts, a = acrocentric, m = metacentric, sm = submetacentric; inversions (i); and fusions between two chromosomes (-).

Given a well-resolved phylogeny, we can now investigate the history of chromosome change in the genus. Using parsimony mapping of CRs, as identified by G-banding (Eldridge and Close, 1993) we were able to resolve ancestral nodes where rearrangements occurred, in particular a single origin of CR 7a (a = acrocentric). However, for some chromosomes, we could not distinguish between different hypotheses (see **Figure 3**; Supplementary Table 2). Apparent multiple independent origins of the 3a, 4a, 4sm (sm = submetacentric) and 5i (i = inversion) rearrangements suggest there could be regions of the genome susceptible to rearrangement processes (“hotspots”), which have also been implicated in chromosome change in other macropodids (Bulazel et al., 2007). This highlights the potential for convergent evolution of rearrangements, including chromosomal fusions (e.g., 6 and 10 fusion), inversions and centromere shifts. The alternate hypotheses are multiple reversals to an ancestral chromosome morphology, or the introgression of chromosomes between taxa, but further analysis (e.g., sequencing of breakpoints) is required to distinguish between them. In addition to parallel evolution, the same chromosomes are involved in fusion events in different taxa (e.g., 5, 6, 9, and 10). Cell culture experiments using mitomycin C to induce centric fusions showed that chromosome 10 fused most frequently (Eldridge and Johnston, 1993). Despite all chromosomes being involved in fusions in this experiment, the higher frequency of chromosome 10 fusions *in vitro* matches the larger proportion of chromosome 10 being involved in fusions in the wild in *Petrogale* (five out of eight fusions). This together with higher frequencies of breakpoints from gamma radiation in chromosomes 5, 6 and 10 (Eldridge and Johnston, 1993) further support the notion of a nonrandom process of CR.

**FIGURE 4:**
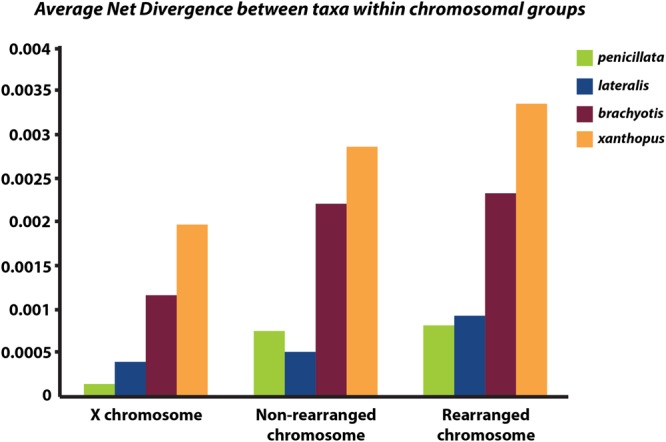
**Graph of average net divergence between taxa within each chromosomal group: *brachyotis, lateralis, penicillata*, and *xanthopus.*** Divergences are estimated for loci on the X chromosome, non-rearranged chromosomes (2,4,7,8) and rearranged chromosomes (5,6,9,10).

Next, we partitioned the sequenced exons into autosomal-non-rearranged (*N* = 140 exons; 75,296 bp), autosomal-rearranged (*N* = 160 exons; 36,168 bp), and the X chromosome (*N* = 21 exons; 8,951 bp). This approach was motivated by the expectation that autosomes will have less phylogenetic signal than the X because of their higher gene flow rates and larger *N*_e_ (see Supplementary Table 3 for mapped loci). Mean divergence between the four chromosomal groups varies across the X chromosome, rearranged and non-rearranged autosomes (**Figure 4**). When accounting for differences in sequence length, we find that the X has reduced diversity compared with the autosomes, although only slightly compared to the rearranged autosomes. In other mammalian systems (e.g., apes – Nam et al., 2014) several selective sweeps have created large regions of low diversity on the X chromosome. Assessment of more loci along the X is necessary to explore if the lower diversity on the X within *Petrogale* is associated with selection. The effects of small *N*_e_ on the X will need to be assessed to distinguish between selective sweeps and neutral models of evolution. However, given that sex chromosomes contribute disproportionately to post-zygotic isolation in many taxa (e.g., *Drosophila*, Presgraves, 2008; Flycatchers, Saether et al., 2007), and evidence of the *Petrogale* system conforming to Haldane’s rule, we would expect more loci on the X to conform to the true species phylogeny than autosomal loci (as argued by Fontaine et al., 2015).

**FIGURE 5:**
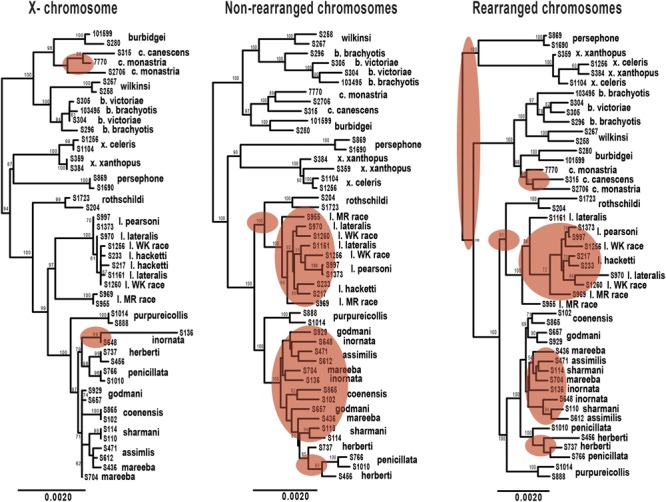
**Maximum likelihood phylogenies of *Petrogale* taxa based on loci from the X chromosome, rearranged autosomal chromosomes (5,6,9,10) and non-rearranged autosomal chromosomes (2,4,7,8).** Bootstrap support is highlighted on the nodes of the tree. Incongruence in the phylogenetic reconstruction to the concatenated nuclear phylogeny (**Figure 2A**) is highlighted in red on each tree.

Rearranged and non-rearranged chromosomes show similar levels of divergence in the *brachyotis* and *penicillata* groups, which are among the most chromosomally diverse *Petrogale* (**Figure 5**). Conversely, rearranged chromosomes showed the greatest increase in divergence relative to non-rearranged chromosomes in the *lateralis* group, which has relatively few rearrangements. These observations do not tend to support the hypothesis that CRs are a primary cause of speciation. Instead, the CRs may have fixed when species were already isolated. Alternatively, the existing data may be inadequate to distinguish between these hypotheses. Further work is needed.

We expect clearer phylogenetic resolution in rearranged than in non-rearranged regions of the genome, particularly within the *penicillata* group (**Figure 5**). Neither autosomal phylogenetic reconstructions are able to resolve the relationships of the *lateralis* and *penicillata* groups. The phylogeny based on rearranged regions does however, separate *P. herberti* and *P. penicillata* from the remaining taxa, as well as *P. coenensis* and *P. godmani*, compared to the non-rearranged chromosomes. Both autosomal phylogenies resolve the *brachyotis* and *xanthopus* groups, but the rearranged phylogeny places the *xanthopus* group as basal instead of the *brachyotis* group. By contrast, the X loci resolve nodes deeper in the tree but appears to lack enough information to resolve all of the internal relationships of the *lateralis* and *penicillata* groups. The X however, does generally group individuals of a taxon together, unlike the autosomes (**Figure 2**).

We then asked how often the individuals sampled from each species formed a monophyletic group for the different subsets of exons. If rearrangements result in reduced introgression, we expect to see higher concordance in concatenated loci from rearranged than non-rearranged chromosome arms. Further, as the X chromosome is frequently found to be resistant to gene flow, we also expected higher congruence across the X-linked loci. On average, the rearranged chromosomes have greater monophyly of taxa than the non-rearranged for the *penicillata* group, supporting our hypothesis of higher concordance. We do, however, find the opposite pattern for the *lateralis* group (Supplementary Table 4). This may be because the *lateralis* group has fewer rearranged loci. Overall it had lower concordance of monophyletic individuals compared to the *penicillata* group. The X chromosome had the greatest average monophyly. This may result from a smaller *N*_e_ of the X, faster divergence of the X (e.g., Charlesworth et al., 1987), or greater divergent selection on the X. The evolution of the sex chromosomes needs further investigation.

Analysis of all mitochondrial coding genes (12 genes; 11,373 bp), albeit still a single linkage group, reveals some strong conflicts between mitochondrial and nuclear evolutionary history (**Figure 2**). Previously, it has been highlighted that introgression, retained ancestral polymorphism (or ILS) has resulted in paraphyletic species complexes within both the *brachyotis* group (see Potter et al., 2012a,b) and the *penicillata* group (Briscoe et al., 1982; Bee and Close, 1993; Potter et al., 2015). This is the first analysis using all coding genes across the mitochondrial genome, providing the most phylogenetic information and highlight discrepancies to the nuclear phylogeny, in particular – the placement of *P. persephone* and *P. xanthopus* as basal branches; the paraphyly of *brachyotis* group taxa; and lack of monophyly for *P. assimilis, P. coenensis* and *P. godmani* within the *penicillata* group. These inconsistencies further highlight areas of potential reticulation in the history of *Petrogale*; in particular, the potential for repeated episodes of introgression in the history of the genus.

**FIGURE 6:**
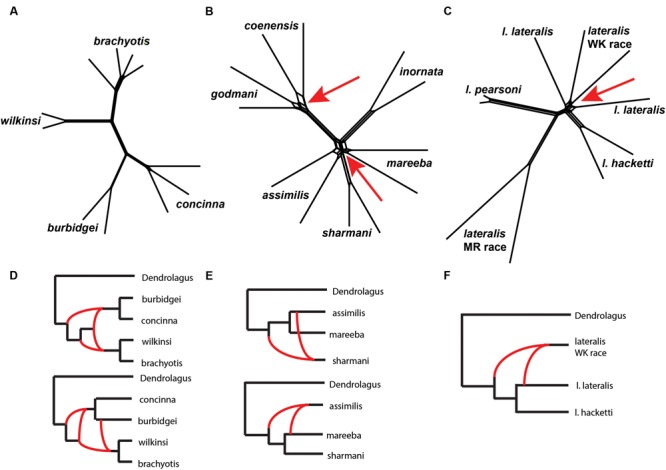
**(A–C)** A phylogenetic network analysis of the chromosomal groups **(A)** *brachyotis*, **(B)** *lateralis*, and **(C)** *penicillata* estimated using a distance based approach in SplitsTree (Neighbor-Net). The red arrows highlight regions on the network where reticulation is inferred. **(D–F)** Model based analysis of reticulate evolutionary history based on analysis of 0–3 reticulations. The lowest log likelihood results are shown for each **(D)** *brachyotis*, **(E)** *lateralis*, and **(F)** *penicillata* chromosomal groups using PhyloNet. Analysis was performed on a single individual for each taxon and two replicate analyses, each including one of the two independent samples per taxon. **(D)** The *brachyotis* group supported 2–3 reticulations and highlight reticulation involving ancestors in the *brachyotis* group. **(E)** The *penicillata* group analysis include a three species complex (*P. assimilis, P. mareeba*, and *P. sharmani*) and support a single reticulation model but alternate topologies and individuals involved in reticulation based on the individuals used in the analysis. **(F)** The *lateralis* group analyses included three taxa (*P. l. lateralis, P. l. hacketti* and *P. l.* West Kimberley race). The results were congruent in identifying a single reticulation model, which involved the *P. lateralis* West Kimberley race.

We then explored patterns of reticulation among chromosomal groups. We based these analyses on concatenated nuclear loci, effectively ignoring coalescent variance in gene trees. We first used an exploratory statistical approach (Neighbor-Net in Splits tree – Bryant and Moulton, 2002; Huson and Bryant, 2006, based on average distances). The results suggested reticulation amongst *P. coenensis* and *P. godmani*, between *P. assimilis, P. mareeba* and *P. sharmani*, as well as between *P. lateralis lateralis* and *P. lateralis* West Kimberley race (**Figure 6**). The *brachyotis* group does not indicate any strong evidence of reticulation between species. The *penicillata* group results match previous results of microsatellites and mtDNA, which inferred gene flow between these taxa, even those with complex CRs (Potter et al., 2015).

We next used an approach based on the multispecies network coalescent (PhyloNet – Than et al., 2008; Yu et al., 2013, 2014). We find evidence of reticulation for all three chromosomal groups (**Figure 6**; Supplementary Table 5). Based on our analysis of up to three reticulation events, the results support 2–3 reticulation nodes in the *brachyotis* group, which suggests historical introgression may explain the discordance between mtDNA and nuclear loci. This included reticulation at nodes of ancestral branches in the *brachyotis* group. For both the *lateralis* and *penicillata* comparisons there was support for a single reticulation node. Within the *lateralis* group, the reticulation node involved *P. lateralis* West Kimberley race. Reticulation could reflect ILS between *P. l. lateralis* and *P. l. hacketti* or introgression with *P. l. lateralis*. In the *penicillata* group, the analyses were less concordant, and the reticulation node included *P. assimilis* and *P. sharmani* for the independent analysis. Both network analyses reflect greater reticulation across all three species and include introgression with *P. mareeba* and *P. assimilis*, as well as ILS amongst the three species. In all three chromosomal group comparisons, reticulation is evident between species with CRs (fusions and centric shifts). Further work is necessary to disentangle the effects of ILS from introgression, which will require further sampling in rearranged vs. non-rearranged regions of chromosomes.

Understanding the genome, the physical position of loci and how they interact is crucial in interpreting the evolutionary history of organisms. Our results highlight that if certain loci taken alone without any context of chromosome structure can yield completely different results and a misunderstanding of the mechanisms involved in reproductive isolation. We are still in the early stages of understanding the physical location of loci in this non-model system and as further work allows mapping of loci to chromosomes and regions of rearrangements, we will be better able to test for recombination suppression and reduced gene flow in these regions compared to the non-rearranged chromosomes. With the addition of X chromosome loci, we may be able to further understand the genetic variation on the X which itself has a high rate of intrachromosomal rearrangement in this system. The combined effects of faster X divergence and reduced recombination may affect speciation in this genus. Below, we outline the necessary steps to progress this non-model system – a framework we believe useful for any non-model speciation research.

## Prospectus

The case study above, for a fascinating yet “non-model” system, indicates both the promise and the challenges of arriving at a holistic understanding of how CRs affect divergence and adaptation. Hybrid infertility and recombination suppression (adaptive) models have largely been treated as exclusive, but in systems with complex rearrangements, both could well be in play (Faria and Navarro, 2010; Garagna et al., 2014). Further, such hypotheses should not be treated as exclusive of accumulation of genic incompatibilities, such as large X-effects (Presgraves, 2008), effects of recessive X-linked incompatibility alleles on sterility of the heterogametic sex (Haldane’s rule; Haldane, 1922; Turelli, 1998; Presgraves, 2008, 2010) or cytonuclear incompatibilities (Turelli and Moyle, 2007). In addition to new evidence across diverse systems, we need further development of theory that incorporates these co-occurring processes (Feder and Nosil, 2009; Faria and Navarro, 2010). We should also consider extended models with meiotic drive, genomic conflict and disruption of epigenetic programming (Brown and O’Neill, 2010).

**FIGURE 7:**
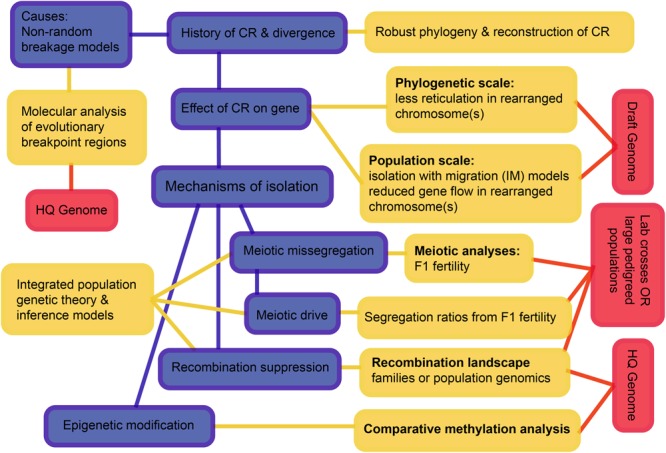
**A schematic overview of an integrative approach to increase understanding how chromosomal rearrangements create divergence and speciation.** The hierarchy outlined progresses from simple to complex and may not be feasible for all non-model systems, but outlines the ultimate goals. Blue boxes highlight the processes, yellow boxes highlight the analyses and red boxes highlight the data needed to achieve a holistic understanding of chromosomal speciation. CR, chromosome rearrangement; HQ, high quality.

Considering all these interacting processes, we suggest a hierarchy of questions and associated requirements that progress from simple to challenging (**Figure 7**). The first question is the history of speciation and CRs, as we have now begun to resolve for the rock-wallabies. We move on to testing the effects of CRs on the suppression of gene flow, and then to testing mechanisms that may have suppressed gene flow. The scale and diversity of data needed expands as we move from pattern to process. Of particular note is the need to develop new theory and inference methods that can distinguish the different processes by integrating genomic data, recombination landscapes, meiotic irregularities, segregation patterns, and epigenetics. Such methods involve complicated parameter estimation and may need to rely on approaches such as approximate Bayesian computation (ABC) to compare competing models (see Beaumont, 2010). Those models, in turn, will likely employ multispecies coalescence that includes speciation and demographic history, as well as the evolution of CRs (e.g., coalescent models for CRs – Guerrero et al., 2012a,b; Ayala et al., 2013; Peischl et al., 2013). The quality of genome assemblies needed increases as we move from pattern to process. This is crucial to remove errors that can influence inference of rearrangements as well as recombination rate changes.

In the following, we explore three key issues that have emerged: (i) what do we know of differential origins of new (or recurrent) CRs; (ii) how do CRs interact with the epigenetic programming of genes and chromosome segments; and (iii) what new theory and inference methods do we need to exploit comparative genomic data in the context of individual and combined effects of CRs.

### Origins of CRs

Recent reviews highlight that the breakpoints of CRs are associated with repetitive elements, and are influenced by functional constrains and meiotic recombination (Farré et al., 2015). These features have been discovered from whole genome comparisons of distantly and closely related species. Repetitive sequences, such as segmental duplications and transposable elements, appear to provide the substrates for non-allelic homologous recombination (Bailey et al., 2004; Cordaux and Batzer, 2009), resulting in CRs such as inversions. These breakpoint regions are also common in gene-dense regions of the genome, with the breaks occurring predominantly in intergenic regions where they are less likely to silence a gene (Lemaitre et al., 2009). Breakpoint regions are enriched with genes involved in adaptive processes, such as immune response genes, and CRs causing changes to expression of these genes or otherwise rendering a gene non-functional could provide a selective advantage to result in the fixation of this rearrangement (Larkin et al., 2009; Ullastres et al., 2014). Furthermore, gene-rich regions have been shown to be more actively transcribed, possessing epigenetic characteristics of open, and therefore accessible, chromatin (Lemaitre et al., 2009; Capilla et al., 2016). This makes sense in light of the observation that meiotic double strand breaks required for CRs typically occur in transcriptionally active, open chromatin regions of the genome (Smagulova et al., 2011).

Rearrangements involving centromeres are common in many systems, including *Petrogale*. Similar to the breakpoint regions described above, centromeres contain highly repetitive sequences. Robertsonian fusions may be formed from illegitimate recombination occurring among these highly repetitive sequences (Slijepcevic, 1998; Garagna et al., 2001; Ruiz-Herrera et al., 2006). Nuclear architecture also appears to play a role in the formation of Robertsonian fusions. In mice, the pericentric regions of telocentric chromosomes converge during the leptotene stage of prophase I in meiosis, placing pericentromeric regions of different chromosomes in close proximity (Garagna et al., 2014). Prophase I is also the time when DNA is damaged and the cell is repairing the double-stranded breaks by homologous recombination for synapsis of homologous chromosomes (Neale and Keeney, 2006). The combination of these two factors lends this phase of meiosis to being a time of potential CR (Garagna et al., 2014). More generally, the location of chromosomes within the nucleus should be considered when studying CRs as it could help to understand why some chromosomes or chromosome arms more commonly involved in CRs than other chromosomes (Wesche and Robinson, 2012).

In some cases, the CR involving the centromere is a centric shift. This could occur via a pericentric inversion. Alternatively, neocentromeres may be established from epigenetic changes to repetitive sequences located elsewhere on the chromosome and old centromeres decommissioned (O’Neill et al., 2004). Also, a three-break rearrangement that allows the centromere to be excised and then reinserted in a different position further along the chromosome could occur (Eldridge et al., 1988). O’Neill et al. (2004) suggested that heterochromatin stabilizes centromere position and its absence opens the way for neocentromerization. Although cause and consequence remain difficult to distinguish, and further exploration of this hypothesis in *Petrogale* would be of value.

The characterization of breakpoint features and the role of centromeres in CRs has made it evident that sequence content, an open chromatin conformation and chromosome territories within the nucleus in the germline are all important for understanding the origin of CRs and should be considered together (i.e., the “Integrative Breakage Model,” Farré et al., 2015). Detailed studies of epigenomic features and chromatin conformation are possible for model species like the mouse (Capilla et al., 2016), testing the Integrative Breakage Model may be more challenging at present for non-model species as a high-quality reference genome is essential for interpreting epigenomic data. However, advances in sequencing technology and the corresponding genome assembly pipelines (e.g., Putnam et al., 2016), make this achievable for non-model species.

### Interaction of CRs with Epigenetic Programming

Epigenetic variation has recently been recognized to cause genetic incompatibilities that can lead to reproductive isolation (Brown and O’Neill, 2010; Durand et al., 2012). The only known “speciation gene” in mammals is *Prdm9* in mice (Mihola et al., 2009). It encodes for the meiotic specific-protein responsible for marking the location of recombination hotspots (Grey et al., 2011). Recombination is essential recognition of chromosome homologues during prophase I of meiosis and its disruption results in sterile male hybrids. An interaction between the autosomal *Prdm9* and the X-linked *Meir1* gene contributes to reproductive isolation of *Mus musculus musculus* and *Mus m. domesticus* subspecies (Balcova et al., 2016). Robertsonian fusions alter the epigenetic marks of H3K9me3 and γH2AX (Capilla et al., 2014). The accumulation of γH2AX is associated with the MSUC mechanism, which is similar to meiotic sex chromosome inactivation (MSCI) for silencing the unsynapsed X and Y in males. These mechanisms involve a suite of epigenetic modifications to achieve transcriptional silencing of the sex chromosomes (MSCI) or unsynapsed region (MSUC) (reviewed in Turner, 2007). When CRs reduce recombination, MSUC may therefore silence genes critical to meiosis (Shiu et al., 2001) and cause infertility (Garagna et al., 2014).

The frequent involvement of centromeres in CRs makes it interesting to consider the role of these important structures in speciation. Sequences at centromeres rapidly evolve, differing markedly between closely related species. Chromatin-binding proteins, such as various histone proteins, are important for the normal function of a centromere during meiosis and mitosis. Reproductive isolation could result from incompatibilities in these proteins, with chromatin-binding proteins from one species failing to recognize the repeat sequence of the other (Sawamura et al., 2012). Centromere incompatibilities could then lead to centromere-drive where there is an imbalance in centromere strength during meiosis, contributing to post-zygotic isolation (Henikoff et al., 2001; Malik and Henikoff, 2003).

### Population Genetics – Theory and Inference

Substantial progress has been made toward understanding speciation using sequence data. Faria and Navarro (2010) develop a framework based on the models of Navarro and Barton (2003) and Kirkpatrick and Barton (2006) to study how CRs contribute to speciation and how they first become fixed in different populations. This links population genetics theory to speciation and encompasses a model of how CRs have become fixed outside of previous models based on drift (Walsh, 1982; Lande, 1985; Spirito, 1998), selective advantages (Nei et al., 1967), meiotic drive (Nei et al., 1967), or epistatic interactions (Charlesworth, 1974).

There is the need for development of models that incorporate synergistic effects of genic and chromosomal variation, as well as the effects of drift and selection. Such models might provide opportunities to evaluate when a single process can explain observed patterns of variation in a dataset, and when multiple interacting processes need to be invoked. However, the reality is that genic and chromosomal divergences occur in parallel, CRs potentially have multiple effects, and that such models will only be capable of distinguishing among hypothesized processes in the simplest of systems. Here comparative genomic datasets, in particular lineages with a history of recent CRs, might offer unique opportunities. With such data, it is possible to test predictions of molecular evolution and biogeography, but empirical data can produce similar signatures under different scenarios (Noor and Bennett, 2009). One possible way forward is to exploit the different types of evidence (**Figure 7**) to set up competing/complementary models of single and joint effects that could then be evaluated using comparative sequence data.

In systems with multiple CRs, like the *Petrogale* system described here, we can view each CR as a semi-independent evolutionary replicate, providing opportunities to examine how different independent CRs, fixing in populations at different times, relate to genic divergence. This in turn can inform our understanding of processes. For instance, evidence of synchrony in fixation of different rearrangements may provide evidence of meiotic drive (e.g., Pardo-Manuel de Villena and Sapienza, 2001). Under meiotic drive hypotheses, we expect rearrangements will be of similar ages. If, however, CRs generate beneficial fitness effects (spread by positive selection), we do not expect fixation to occur at similar times. With combined cytogenetic understanding, this allows us to fit models to different regions along each chromosome to capture their unique evolutionary histories. If rearrangements are important to divergence, we expect the times at which they are established to coincide with speciation events. Using coalescent models to date speciation events and then mapping rearrangements on these phylogenies will help elucidate which CRs could have been involved in speciation (also previously suggested by Faria and Navarro, 2010). The key here will be finding systems where CRs are relatively recent, to distinguish between genic divergence post speciation vs. mutations causing reproductive isolation in relation to genomic architecture.

## Conclusion

Integration of cytogenetic, genomic, and epigenetic data using a holistic approach will be crucial to improving our understanding of how genomic architecture influences and potentially drives reproductive isolation amongst organisms. The non-model system highlighted here has value for many reasons aside from its recent origin and chromosomal diversity. These include: the effect of reversed recombination rate between the sexes in marsupials relative to most other eutherians (males > females), X and Y chromosomes do not pair during meiosis (they lack the pseudoautosomal region), and epigenetic mechanisms vary significantly from eutherians. This one non-model system illustrates how biological variation can provide valuable contrasts to model systems. The growth of genomic and computational technology is opening new vistas on fundamental questions about how genomic architecture influences evolution.

## Data Accessibility

Raw sequencing reads associated with this study are available at NCBI Sequence Read Archive (http://www.ncbi.nlm.nih.gov/sra; BioProject PRJNA360868; BioSamples SAMN06233288-SAMN06233338). A Dryad Repository (http://dx.doi.org/10.5061/dryad.mm856) contains code that was used in data analysis and Sequence Read Archive accession details.

## Ethics Statement

This study did not use live animals and therefore does not require animal ethics approval. Samples used were tissues from the Australian Museum collection. Animal research at the Australian Museum and in Australia is governed by the “Australian code for the care and use of animals for scientific purposes.” The Code only requires a research project to be approved by an ethics committee if the research involves live animals.

## Author Contributions

SP had substantial contribution to the conception, acquisition, design, analysis and interpretation of the work, drafting the work, final approval of the version to be published and agreement to be accountable for all aspects of the work. JB had substantial contribution to the conception, design, analysis and interpretation of the work, drafting the work, final approval of the version to be published and agreement to be accountable for all aspects of the work. MB had substantial contribution to the analysis of the work, drafting the work, final approval of the version to be published and agreement to be accountable for all aspects of the work. JD had substantial contribution to the conception, acquisition and design of the work, drafting the work, final approval of the version to be published and agreement to be accountable for all aspects of the work. MK had substantial contribution to the conception of the work, drafting the work, final approval of the version to be published and agreement to be accountable for all aspects of the work. ME and CM had substantial contribution to the conception, acquisition, design and interpretation of the work, drafting the work, final approval of the version to be published and agreement to be accountable for all aspects of the work.

## Conflict of Interest Statement

The handling Editor declared a shared affiliation, though no other collaboration, with one of the authors JD and states that the process nevertheless met the standards of a fair and objective review. The other authors declare that the research was conducted in the absence of any commercial or financial relationships that could be construed as a potential conflict of interest.
