# Supplementary material for: Chromosomal Speciation in the Genomics Era: Disentangling Phylogenetic Evolution of Rock-wallabies
Source: Front Genet. 2017 Feb 10;8:10. doi: 10.3389/fgene.2017.00010 (PMC5301020; doi:10.3389/fgene.2017.00010)
Supplement: Supplementary file 1 [file Table_1.docx]

**Supplementary Table 1** Taxon information for each individual, including: sample ID, species/sub-species/race, locality, latitude and longitude and index number for the nuclear exon capture experiments.

| **Sample ID** | **Tissue ID** | **Museum Voucher** | **Species** | **Locality** | **State** | **Latitude** | **Longitude** | **Index** |
| --- | --- | --- | --- | --- | --- | --- | --- | --- |
| S471 |  | CM12634 | *assimilis* | Charters Towers | Queensland | -20.26 | 146.01 | SP12_indexing29 |
| S612 |  | CM13533 | *assimilis* | Hillsborough | Queensland | -20.10 | 147.04 | SP12_indexing30 |
| S296 |  | CM15326 | *brachyotis brachyotis* | Beverley Springs | Western Australia | -16.51 | 125.33 | SP12_indexing47 |
| ABTC103495 |  | none | *brachyotis brachyotis* | Monsmont Island | Western Australia | -16.30 | 128.70 | SP12_indexing48 |
| S304 | EBU35576 | CM15306 | *brachyotis victoriae* | Lobby Cr, Bradshaw | Northern Territory | -15.33 | 130.10 | SP12_indexing45 |
| S305 |  | none | *brachyotis victoriae* | Goobaieri Bay, Bradshaw | Northern Territory | -15.20 | 129.85 | SP12_indexing46 |
| ABTC101599 |  | none | *burbidgei S* | Prince Regent River | Western Australia | -15.63 | 125.26 | SP12_indexing40 |
| S280 |  | CM15326 | *burbidgei N* | Crystal Cr., Mitchell Plateau | Western Australia | -14.50 | 125.79 | SP12_indexing41 |
| S102 |  | none | *coenensis* | Coen | Queensland | -13.83 | 143.13 | SP12_indexing36 |
| S865 |  | CM13479 | *coenensis* | Musgrave | Queensland | -14.80 | 143.43 | SP12_indexing37 |
| S315 | EBU35578 | none | *concinna canescens* | Mt Borrodaile | Northern Territory | -12.05 | 132.90 | SP12_indexing1 |
| ABTC7770 |  | WAM23036 | *concinna monastria* | Hidden Island | Western Australia | -16.25 | 123.62 | SP12_indexing2 |
| S2706 |  | none | *concinna monastria* | Augustus Island | Western Australia | -15.35 | 124.55 | SP12_indexing52 |
| S657 |  | CM13466 | *godmani* | Bathurst Heads | Queensland | -14.31 | 144.21 | SP12_indexing38 |
| S929 |  | CM16827 | *godmani* | Mt Mulgrave | Queensland | -16.44 | 144.13 | SP12_indexing39 |
| S456 |  | CM12619 | *herberti* | Sebastpol Hill, Westwood | Queensland | -23.65 | 150.15 | SP19_indexing25 |
| S737 |  | CM13506 | *herberti* | Mt Donneybrook | Queensland | -22.51 | 146.74 | MP01_indexing53 |
| S648 |  | none | *inornata* | Gregory Range | Queensland | -19.93 | 147.32 | SP12_indexing33 |
| S136 |  | CM15211 | *inornata* | Maiden Mountain | Queensland | -19.93 | 147.87 | SP12_indexing34 |
| S233 |  | CM15381 | *lateralis hacketti* | Wilson Island, Recherche Archipelago | Western Australia | -34.12 | 122.00 | SP12_indexing13 |
| S217 |  | CM15310 | *lateralis hacketti* | Westall Island, Recherche Archipelago | Western Australia | -34.08 | 122.97 | SP12_indexing14 |
| S970 |  | CM16823 | *lateralis lateralis* | Nangeen Hill | Western Australia | -31.83 | 117.68 | SP12_indexing17 |
| S1161 |  | none | *lateralis lateralis* | Barrow Island | Western Australia | -20.77 | 115.33 | SP12_indexing18 |
| S969 | EBU47377 | AM29508 | *lateralis* MacDonnell Ranges race | Heavitree Gap, Alice Springs | Northern Territory | -23.74 | 133.87 | SP12_indexing23 |
| S955 |  | CM24567 | *lateralis* MacDonnell Ranges race | Telegraph Station, Alice Springs | Northern Territory | -23.67 | 133.88 | SP12_indexing24 |
| S997 |  | CM24572 | *lateralis pearsoni* | Wedge Island | South Australia | -35.16 | 136.46 | SP12_indexing15 |
| S1373 | EBU47375 | none | *lateralis pearsoni* | South Pearson Island | South Australia | -33.95 | 134.27 | SP12_indexing16 |
| S1256 | EBU47376 | none | *lateralis* West Kimberley race | Erskine Range, West Kimberley | Western Australia | -17.81 | 124.33 | SP12_indexing11 |
| S1260 |  | none | *lateralis* West Kimberley race | Logues Springs, West Kimberley | Western Australia | -18.42 | 123.08 | SP12_indexing12 |
| S704 |  | CM13473 | *mareeba* | Walsh River Gorge | Queensland | -16.88 | 143.95 | SP12_indexing25 |
| S436 |  | CM10492 | *mareeba* | Anthill Creek | Queensland | -18.39 | 145.14 | SP12_indexing26 |
| S766 | EBU35579 | CM16835 | *penicillata* | Jenolan Caves | New South Wales | -33.82 | 150.03 | SP12_indexing19 |
| S1010 |  | none | *penicillata* | Rocky Plains Creek, East Gippsland | Victoria | -36.91 | 148.24 | SP12_indexing20 |
| S869 | EBU47379 | AM37280 | *persephone* | Gloucester Island | Queensland | -20.04 | 148.45 | SP12_indexing21 |
| S1690 |  | none | *persephone* | Mt Lucas | Queensland | -20.34 | 148.58 | SP12_indexing22 |
| S888 | EBU47380 | CM16832 | *purpureicollis* | Dajarra | Queensland | -21.67 | 139.29 | SP12_indexing9 |
| S1014 |  | none | *purpureiciollis* | Lawn Hill | Queensland | -18.67 | 138.33 | SP12_indexing10 |
| S204 | EBU47381 | CM15324 | *rothschildi* | Rosemary Island, Dampier Archipelago | Western Australia | -20.49 | 116.59 | SP12_indexing3 |
| S1723 |  | WAM51607 | *rothschildi* | Enderby Island, Dampier Archipelago | Western Australia | -20.58 | 116.56 | SP12_indexing4 |
| S114 |  | none | *sharmani* | Mt. Claro | Queensland | -18.87 | 145.73 | SP12_indexing27 |
| S110 |  | CM15203 | *sharmani* | Mt. Claro | Queensland | -18.87 | 145.73 | SP12_indexing28 |
| S267 |  | CM15172 | *wilkinsi* | Butterfly Gorge, Douglas R. | Northern Territory | -13.75 | 131.58 | SP12_indexing43 |
| S258 |  | CM15311 | *wilkinsi* | Roper River (Ngukurr) | Northern Territory | -15.05 | 134.68 | SP12_indexing44 |
| S1104 |  | none | *xanthopus celeris* | Captive, QPWS, Charleville | Queensland | - | - | SP12_indexing5 |
| S1157 | EBU47383 | none | *xanthopus celeris* | Captive, QPWS, Charleville | Queensland | - | - | SP12_indexing6 |
| S359 | EBU47382 | none | *xanthopus xanthopus* | Middle Gorge, Quorn | South Australia | -32.17 | 138.04 | SP12_indexing7 |
| S384 | RW307 | CM05793 | *xanthopus xanthopus* | Cootawundi Station | South Australia | -31.05 | -142.08 | SP12_indexing8 |
| S1475 |  | none | *Dendrolagus lumholtzi* | Old Palmerston Hwy, Atherton Tablelands | Queensland | -17.56 | 145.61 | SP12_indexing53 |
| S1469 |  | none | *Dendrolagus lumholtzi* | Thomas Rd via Yungaburra | Queensland | -17.26 | 145.56 | SP12_indexing54 |
| S1175 |  | AM37274 | *Thylogale thetis* | Gumleaf Hut, Chickester State Forest | Queensland | -32.27 | 151.75 | SP12_indexing55 |
| S1033 |  | none | *Thylogale thetis* | O'Reillys, Lamington National Park | Queensland | -28.23 | 153.13 | SP12_indexing56 |
