# Supplementary material for: Chromosomal Speciation in the Genomics Era: Disentangling Phylogenetic Evolution of Rock-wallabies
Source: Front Genet. 2017 Feb 10;8:10. doi: 10.3389/fgene.2017.00010 (PMC5301020; doi:10.3389/fgene.2017.00010)
Supplement: Supplementary file 2 [file Table_2.docx]

**Supplementary Table 2** Character state matrix of chromosomal rearrangements for the *Petrogale* genus. Ancestral characters are given state 0, whilst alternatives are given states 1-5. This matrix was used in phylogenetic reconstruction of chromosomal evolution.

**Character State Reference**

| **0 (ancestral state)** | **1** | **2** | **3** | **4** | **5** | **6** | **7** | **8** | **9** | **10** |
| --- | --- | --- | --- | --- | --- | --- | --- | --- | --- | --- |
| **1** | 1-10 | 2s | 3a | 4a | 5i | 6-10 | 7a | 8m | 9-10 | 6-10 |
| **2** |  |  | 3a-6 | 4sm | 5-9 | 6-10a |  |  | 5-9 | 6-10a |
| **3** |  |  |  | 4s | 5s | 6-9a |  |  | 6-9a | 9-10 |
| **4** |  |  |  |  | 5-10 | 3a-6 |  |  |  | 5-10 |
| **5** |  |  |  |  |  |  |  |  |  | 1-10 |
|  |  |  |  |  |  |  |  |  |  |  |
|  |  |  |  |  |  |  |  |  |  |  |
| **Character matrix** |  |  |  |  |  |  |  |  |  |  |
|  | **1** | **2** | **3** | **4** | **5** | **6** | **7** | **8** | **9** | **10** |
| ***assimilis*** | 0 | 0 | 1 | 1 | 1 | 1 | 1 | 0 | 0 | 1 |
| ***burbidgei*** | 1 | 1 | 1&2 | 2 | 2 | 4 | 0 | 1 | 2 | 5 |
| ***brachyotis*** | 1 | 1 | 1&2 | 2 | 3 | 4 | 0 | 0 | 0 | 5 |
| ***coenensis*** | 0 | 0 | 1 | 0 | 0 | 0 | 1 | 0 | 0 | 0 |
| ***concinna canescens*** | 1 | 1 | 1&2 | 0 | 2 | 4 | 0 | 1 | 2 | 5 |
| ***godmani*** | 0 | 0 | 1 | 1 | 1 | 1&2 | 1 | 0 | 0 | 1&2 |
| ***herberti*** | 0 | 0 | 1 | 3 | 0 | 0 | 1 | 0 | 0 | 0 |
| ***inornata*** | 0 | 0 | 1 | 0 | 1 | 0 | 1 | 0 | 0 | 0 |
| ***lateralis hacketti*** | 0 | 0 | 0 | 0 | 0 | 1 | 0 | 0 | 0 | 1 |
| ***lateralis lateralis*** | 0 | 0 | 0 | 0 | 0 | 0 | 0 | 0 | 0 | 0 |
| ***lateralis pearsoni*** | 0 | 0 | 0 | 0 | 0 | 0 | 0 | 0 | 0 | 0 |
| ***lateralis* MR race** | 0 | 0 | 1 | 0 | 0 | 0 | 0 | 0 | 0 | 0 |
| ***lateralis* WK race** | 0 | 0 | 0 | 0 | 0 | 0 | 0 | 0 | 1 | 3 |
| ***mareeba*** | 0 | 0 | 1 | 1 | 4 | 3 | 1 | 0 | 3 | 4 |
| ***penicillata*** | 0 | 0 | 1 | 1 | 0 | 0 | 1 | 0 | 0 | 0 |
| ***persephone*** | 0 | 0 | 0 | 0 | 0 | 0 | 0 | 0 | 0 | 0 |
| ***purpureicollis*** | 0 | 0 | 1 | 0 | 0 | 0 | 0 | 0 | 0 | 0 |
| ***rothschildi*** | 0 | 0 | 0 | 0 | 0 | 0 | 0 | 0 | 0 | 0 |
| ***sharmani*** | 0 | 0 | 1 | 1 | 4 | 3 | 1 | 0 | 3 | 4 |
| ***wilkinsi*** | 1 | 1 | 1&2 | 2 | 3 | 4 | 0 | 0 | 0 | 5 |
| ***xanthopus*** | 0 | 0 | 0 | 0 | 0 | 0 | 0 | 0 | 0 | 0 |
| ***Thylogale*** | 0 | 0 | 0 | 0 | 0 | 0 | 0 | 0 | 0 | 0 |
