# Supplementary material for: Chromosomal Speciation in the Genomics Era: Disentangling Phylogenetic Evolution of Rock-wallabies
Source: Front Genet. 2017 Feb 10;8:10. doi: 10.3389/fgene.2017.00010 (PMC5301020; doi:10.3389/fgene.2017.00010)
Supplement: Supplementary file 3 [file Table_3.docx]

**Supplementary Table 3** Mapping of exons to scaffolds of the tammar wallaby (*Macropus eugenii*) to identify targets on the X, non-rearranged (2,4,7,8) and rearranged (5,6,9,10) chromosomes based on the 2n = 22 ancestor.

| **Exon** | **Scaffold** | **2n = 22 ancestor** |  | **Exon** | **Scaffold** | **2n = 22 ancestor** |
| --- | --- | --- | --- | --- | --- | --- |
| ENSSHAP00000000053_exon2 | scaffold155 | 1 |  | ENSSHAP00000005803_exon1 | scaffold33 | 4 |
| ENSSHAP00000000053_exon7 | scaffold155 | 1 |  | ENSSHAP00000005803_exon1 | scaffold27 | 4 |
| ENSSHAP00000000088_exon9 | scaffold28 | 1 |  | ENSSHAP00000005966_exon2 | scaffold351 | 4 |
| ENSSHAP00000000183_exon8 | scaffold28 | 1 |  | ENSSHAP00000005994_exon2 | scaffold27 | 4 |
| ENSSHAP00000000211_exon5 | scaffold155 | 1 |  | ENSSHAP00000005996_exon6 | scaffold27 | 4 |
| ENSSHAP00000000211_exon6 | scaffold155 | 1 |  | ENSSHAP00000006085_exon8 | scaffold33 | 4 |
| ENSSHAP00000000255_exon3 | scaffold28 | 1 |  | ENSSHAP00000006104_exon3 | scaffold351 | 4 |
| ENSSHAP00000000584_exon7 | scaffold28 | 1 |  | ENSSHAP00000006245_exon4 | scaffold27 | 4 |
| ENSSHAP00000000624_exon1 | scaffold155 | 1 |  | ENSSHAP00000006307_exon1 | scaffold351 | 4 |
| ENSSHAP00000000648_exon1 | scaffold77 | 1 |  | ENSSHAP00000006728_exon2 | scaffold33 | 4 |
| ENSSHAP00000001073_exon1 | scaffold28 | 1 |  | ENSSHAP00000006775_exon2 | scaffold27 | 4 |
| ENSSHAP00000001642_exon1 | scaffold28 | 1 |  | ENSSHAP00000007257_exon3 | scaffold33 | 4 |
| ENSSHAP00000001654_exon2 | scaffold155 | 1 |  | ENSSHAP00000007274_exon1 | scaffold351 | 4 |
| ENSSHAP00000001654_exon5 | scaffold155 | 1 |  | ENSSHAP00000007490_exon19 | scaffold351 | 4 |
| ENSSHAP00000001987_exon3 | scaffold155 | 1 |  | ENSSHAP00000007690_exon15 | scaffold33 | 4 |
| ENSSHAP00000002068_exon3 | scaffold155 | 1 |  | ENSSHAP00000007907_exon13 | scaffold33 | 4 |
| ENSSHAP00000002255_exon1 | scaffold155 | 1 |  | ENSSHAP00000007907_exon19 | scaffold33 | 4 |
| ENSSHAP00000002255_exon1 | scaffold155 | 1 |  | ENSSHAP00000007985_exon2 | scaffold351 | 4 |
| ENSSHAP00000002255_exon1 | scaffold155 | 1 |  | ENSSHAP00000008316_exon2 | scaffold33 | 4 |
| ENSSHAP00000002274_exon4 | scaffold155 | 1 |  | ENSSHAP00000008414_exon9 | scaffold33 | 4 |
| ENSSHAP00000002509_exon11 | scaffold77 | 1 |  | ENSSHAP00000008632_exon1 | scaffold351 | 4 |
| ENSSHAP00000002509_exon11 | scaffold77 | 1 |  | ENSSHAP00000008781_exon5 | scaffold33 | 4 |
| ENSSHAP00000003126_exon3 | scaffold155 | 1 |  | ENSSHAP00000008847_exon9 | scaffold33 | 4 |
| ENSSHAP00000003131_exon1 | scaffold155 | 1 |  | ENSSHAP00000008928_exon8 | scaffold27 | 4 |
| ENSSHAP00000003517_exon6 | scaffold28 | 1 |  | ENSSHAP00000009454_exon3 | scaffold351 | 4 |
| ENSSHAP00000003678_exon10 | scaffold155 | 1 |  | ENSSHAP00000009523_exon1 | scaffold33 | 4 |
| ENSSHAP00000003792_exon2 | scaffold155 | 1 |  | ENSSHAP00000009668_exon15 | scaffold351 | 4 |
| ENSSHAP00000004068_exon3 | scaffold28 | 1 |  | ENSSHAP00000009668_exon24 | scaffold351 | 4 |
| ENSSHAP00000004445_exon10 | scaffold77 | 1 |  | ENSSHAP00000009689_exon1 | scaffold33 | 4 |
| ENSSHAP00000004775_exon8 | scaffold28 | 1 |  | ENSSHAP00000009810_exon19 | scaffold351 | 4 |
| ENSSHAP00000004846_exon1 | scaffold28 | 1 |  | ENSSHAP00000009810_exon5 | scaffold351 | 4 |
| ENSSHAP00000004861_exon3 | scaffold28 | 1 |  | ENSSHAP00000009987_exon9 | scaffold27 | 4 |
| ENSSHAP00000005049_exon8 | scaffold28 | 1 |  | ENSSHAP00000010123_exon1 | scaffold351 | 4 |
| ENSSHAP00000005392_exon2 | scaffold77 | 1 |  | ENSSHAP00000010253_exon11 | scaffold27 | 4 |
| ENSSHAP00000005431_exon4 | scaffold155 | 1 |  | ENSSHAP00000010288_exon2 | scaffold351 | 4 |
| ENSSHAP00000005444_exon20 | scaffold155 | 1 |  | ENSSHAP00000010299_exon32 | scaffold351 | 4 |
| ENSSHAP00000005449_exon2 | scaffold155 | 1 |  | ENSSHAP00000010871_exon2 | scaffold33 | 4 |
| ENSSHAP00000005594_exon1 | scaffold77 | 1 |  | ENSSHAP00000011194_exon4 | scaffold27 | 4 |
| ENSSHAP00000005698_exon17 | scaffold155 | 1 |  | ENSSHAP00000011194_exon4 | scaffold27 | 4 |
| ENSSHAP00000005782_exon7 | scaffold77 | 1 |  | ENSSHAP00000011238_exon1 | scaffold33 | 4 |
| ENSSHAP00000006385_exon13 | scaffold28 | 1 |  | ENSSHAP00000011461_exon6 | scaffold351 | 4 |
| ENSSHAP00000006553_exon8 | scaffold155 | 1 |  | ENSSHAP00000011542_exon2 | scaffold27 | 4 |
| ENSSHAP00000006630_exon1 | scaffold155 | 1 |  | ENSSHAP00000011838_exon3 | scaffold33 | 4 |
| ENSSHAP00000006655_exon3 | scaffold28 | 1 |  | ENSSHAP00000012385_exon6 | scaffold351 | 4 |
| ENSSHAP00000006669_exon2 | scaffold28 | 1 |  | ENSSHAP00000012458_exon11 | scaffold27 | 4 |
| ENSSHAP00000006740_exon4 | scaffold155 | 1 |  | ENSSHAP00000012464_exon12 | scaffold351 | 4 |
| ENSSHAP00000006780_exon14 | scaffold155 | 1 |  | ENSSHAP00000012464_exon12 | scaffold27 | 4 |
| ENSSHAP00000006854_exon1 | scaffold28 | 1 |  | ENSSHAP00000012614_exon10 | scaffold27 | 4 |
| ENSSHAP00000007314_exon5 | scaffold155 | 1 |  | ENSSHAP00000012708_exon1 | scaffold27 | 4 |
| ENSSHAP00000007498_exon7 | scaffold155 | 1 |  | ENSSHAP00000012910_exon1 | scaffold33 | 4 |
| ENSSHAP00000007893_exon15 | scaffold28 | 1 |  | ENSSHAP00000013189_exon3 | scaffold351 | 4 |
| ENSSHAP00000007949_exon3 | scaffold155 | 1 |  | ENSSHAP00000013324_exon7 | scaffold33 | 4 |
| ENSSHAP00000008068_exon4 | scaffold155 | 1 |  | ENSSHAP00000013324_exon7 | scaffold27 | 4 |
| ENSSHAP00000008427_exon9 | scaffold28 | 1 |  | ENSSHAP00000013383_exon7 | scaffold33 | 4 |
| ENSSHAP00000008668_exon6 | scaffold155 | 1 |  | ENSSHAP00000013494_exon7 | scaffold27 | 4 |
| ENSSHAP00000009227_exon7 | scaffold77 | 1 |  | ENSSHAP00000013858_exon2 | scaffold351 | 4 |
| ENSSHAP00000009277_exon3 | scaffold28 | 1 |  | ENSSHAP00000014213_exon1 | scaffold33 | 4 |
| ENSSHAP00000009360_exon2 | scaffold155 | 1 |  | ENSSHAP00000014307_exon12 | scaffold351 | 4 |
| ENSSHAP00000009553_exon32 | scaffold28 | 1 |  | ENSSHAP00000014845_exon2 | scaffold33 | 4 |
| ENSSHAP00000009553_exon70 | scaffold28 | 1 |  | ENSSHAP00000014845_exon2 | scaffold33 | 4 |
| ENSSHAP00000009760_exon13 | scaffold155 | 1 |  | ENSSHAP00000014845_exon2 | scaffold33 | 4 |
| ENSSHAP00000009909_exon12 | scaffold28 | 1 |  | ENSSHAP00000014922_exon8 | scaffold33 | 4 |
| ENSSHAP00000010261_exon1 | scaffold77 | 1 |  | ENSSHAP00000015027_exon1 | scaffold27 | 4 |
| ENSSHAP00000010461_exon17 | scaffold155 | 1 |  | ENSSHAP00000015031_exon9 | scaffold27 | 4 |
| ENSSHAP00000010846_exon18 | scaffold28 | 1 |  | ENSSHAP00000015748_exon4 | scaffold33 | 4 |
| ENSSHAP00000010880_exon3 | scaffold28 | 1 |  | ENSSHAP00000015795_exon5 | scaffold33 | 4 |
| ENSSHAP00000010965_exon20 | scaffold155 | 1 |  | ENSSHAP00000016604_exon1 | scaffold27 | 4 |
| ENSSHAP00000010973_exon20 | scaffold28 | 1 |  | ENSSHAP00000016604_exon1 | scaffold27 | 4 |
| ENSSHAP00000011056_exon10 | scaffold77 | 1 |  | ENSSHAP00000016604_exon8 | scaffold27 | 4 |
| ENSSHAP00000011216_exon2 | scaffold155 | 1 |  | ENSSHAP00000016993_exon12 | scaffold33 | 4 |
| ENSSHAP00000011238_exon1 | scaffold155 | 1 |  | ENSSHAP00000017074_exon11 | scaffold33 | 4 |
| ENSSHAP00000011238_exon1 | scaffold155 | 1 |  | ENSSHAP00000017151_exon2 | scaffold33 | 4 |
| ENSSHAP00000011238_exon1 | scaffold155 | 1 |  | ENSSHAP00000017151_exon4 | scaffold33 | 4 |
| ENSSHAP00000011266_exon2 | scaffold28 | 1 |  | ENSSHAP00000017478_exon7 | scaffold33 | 4 |
| ENSSHAP00000011291_exon2 | scaffold77 | 1 |  | ENSSHAP00000017572_exon3 | scaffold351 | 4 |
| ENSSHAP00000011551_exon5 | scaffold155 | 1 |  | ENSSHAP00000017611_exon1 | scaffold27 | 4 |
| ENSSHAP00000011607_exon2 | scaffold155 | 1 |  | ENSSHAP00000017613_exon17 | scaffold27 | 4 |
| ENSSHAP00000011661_exon3 | scaffold77 | 1 |  | ENSSHAP00000017629_exon5 | scaffold351 | 4 |
| ENSSHAP00000011803_exon4 | scaffold155 | 1 |  | ENSSHAP00000017655_exon4 | scaffold33 | 4 |
| ENSSHAP00000011805_exon1 | scaffold28 | 1 |  | ENSSHAP00000017885_exon6 | scaffold351 | 4 |
| ENSSHAP00000011940_exon6 | scaffold77 | 1 |  | ENSSHAP00000017904_exon1 | scaffold27 | 4 |
| ENSSHAP00000011997_exon2 | scaffold77 | 1 |  | ENSSHAP00000018006_exon2 | scaffold351 | 4 |
| ENSSHAP00000012212_exon38 | scaffold28 | 1 |  | ENSSHAP00000018150_exon11 | scaffold33 | 4 |
| ENSSHAP00000012440_exon11 | scaffold77 | 1 |  | ENSSHAP00000018229_exon1 | scaffold27 | 4 |
| ENSSHAP00000012464_exon12 | scaffold155 | 1 |  | ENSSHAP00000018301_exon3 | scaffold33 | 4 |
| ENSSHAP00000012697_exon15 | scaffold28 | 1 |  | ENSSHAP00000018440_exon10 | scaffold351 | 4 |
| ENSSHAP00000012768_exon5 | scaffold155 | 1 |  | ENSSHAP00000018499_exon6 | scaffold33 | 4 |
| ENSSHAP00000012888_exon8 | scaffold77 | 1 |  | ENSSHAP00000018719_exon14 | scaffold27 | 4 |
| ENSSHAP00000012893_exon57 | scaffold28 | 1 |  | ENSSHAP00000018797_exon22 | scaffold351 | 4 |
| ENSSHAP00000013027_exon8 | scaffold155 | 1 |  | ENSSHAP00000018951_exon13 | scaffold33 | 4 |
| ENSSHAP00000013032_exon14 | scaffold77 | 1 |  | ENSSHAP00000019008_exon13 | scaffold27 | 4 |
| ENSSHAP00000013223_exon11 | scaffold28 | 1 |  | ENSSHAP00000019101_exon1 | scaffold27 | 4 |
| ENSSHAP00000013223_exon8 | scaffold28 | 1 |  | ENSSHAP00000019634_exon18 | scaffold27 | 4 |
| ENSSHAP00000013324_exon7 | scaffold77 | 1 |  | ENSSHAP00000019750_exon2 | scaffold33 | 4 |
| ENSSHAP00000013344_exon1 | scaffold28 | 1 |  | ENSSHAP00000000988_exon2 | scaffold85 | 5 |
| ENSSHAP00000013440_exon9 | scaffold28 | 1 |  | ENSSHAP00000001193_exon5 | scaffold236 | 5 |
| ENSSHAP00000013783_exon2 | scaffold77 | 1 |  | ENSSHAP00000001457_exon1 | scaffold180 | 5 |
| ENSSHAP00000013814_exon2 | scaffold155 | 1 |  | ENSSHAP00000001487_exon5 | scaffold85 | 5 |
| ENSSHAP00000014046_exon6 | scaffold155 | 1 |  | ENSSHAP00000001531_exon1 | scaffold236 | 5 |
| ENSSHAP00000014096_exon6 | scaffold28 | 1 |  | ENSSHAP00000001732_exon2 | scaffold236 | 5 |
| ENSSHAP00000014116_exon10 | scaffold155 | 1 |  | ENSSHAP00000001732_exon5 | scaffold236 | 5 |
| ENSSHAP00000014201_exon68 | scaffold28 | 1 |  | ENSSHAP00000001996_exon5 | scaffold85 | 5 |
| ENSSHAP00000014446_exon1 | scaffold155 | 1 |  | ENSSHAP00000001998_exon1 | scaffold236 | 5 |
| ENSSHAP00000014575_exon3 | scaffold77 | 1 |  | ENSSHAP00000001998_exon1 | scaffold236 | 5 |
| ENSSHAP00000014754_exon40 | scaffold155 | 1 |  | ENSSHAP00000002037_exon9 | scaffold85 | 5 |
| ENSSHAP00000014880_exon6 | scaffold28 | 1 |  | ENSSHAP00000002166_exon5 | scaffold236 | 5 |
| ENSSHAP00000014959_exon33 | scaffold28 | 1 |  | ENSSHAP00000002441_exon12 | scaffold236 | 5 |
| ENSSHAP00000015062_exon11 | scaffold155 | 1 |  | ENSSHAP00000002455_exon5 | scaffold85 | 5 |
| ENSSHAP00000015154_exon4 | scaffold28 | 1 |  | ENSSHAP00000002512_exon1 | scaffold236 | 5 |
| ENSSHAP00000015221_exon1 | scaffold155 | 1 |  | ENSSHAP00000002732_exon9 | scaffold236 | 5 |
| ENSSHAP00000015332_exon1 | scaffold77 | 1 |  | ENSSHAP00000002749_exon3 | scaffold85 | 5 |
| ENSSHAP00000015628_exon2 | scaffold155 | 1 |  | ENSSHAP00000003063_exon8 | scaffold236 | 5 |
| ENSSHAP00000015782_exon6 | scaffold77 | 1 |  | ENSSHAP00000003094_exon2 | scaffold180 | 5 |
| ENSSHAP00000015798_exon5 | scaffold155 | 1 |  | ENSSHAP00000003094_exon2 | scaffold180 | 5 |
| ENSSHAP00000015888_exon3 | scaffold155 | 1 |  | ENSSHAP00000003132_exon1 | scaffold236 | 5 |
| ENSSHAP00000015953_exon3 | scaffold155 | 1 |  | ENSSHAP00000003185_exon5 | scaffold180 | 5 |
| ENSSHAP00000015980_exon5 | scaffold155 | 1 |  | ENSSHAP00000003328_exon2 | scaffold236 | 5 |
| ENSSHAP00000015980_exon7 | scaffold155 | 1 |  | ENSSHAP00000003451_exon4 | scaffold236 | 5 |
| ENSSHAP00000016023_exon8 | scaffold155 | 1 |  | ENSSHAP00000003824_exon8 | scaffold236 | 5 |
| ENSSHAP00000016057_exon7 | scaffold155 | 1 |  | ENSSHAP00000003842_exon17 | scaffold236 | 5 |
| ENSSHAP00000016160_exon2 | scaffold155 | 1 |  | ENSSHAP00000004067_exon2 | scaffold85 | 5 |
| ENSSHAP00000016176_exon5 | scaffold77 | 1 |  | ENSSHAP00000004198_exon1 | scaffold85 | 5 |
| ENSSHAP00000016185_exon6 | scaffold28 | 1 |  | ENSSHAP00000004515_exon1 | scaffold236 | 5 |
| ENSSHAP00000016204_exon15 | scaffold155 | 1 |  | ENSSHAP00000004695_exon1 | scaffold236 | 5 |
| ENSSHAP00000016235_exon2 | scaffold155 | 1 |  | ENSSHAP00000004735_exon4 | scaffold236 | 5 |
| ENSSHAP00000016323_exon21 | scaffold155 | 1 |  | ENSSHAP00000004891_exon5 | scaffold180 | 5 |
| ENSSHAP00000016328_exon1 | scaffold155 | 1 |  | ENSSHAP00000004922_exon8 | scaffold236 | 5 |
| ENSSHAP00000016428_exon1 | scaffold155 | 1 |  | ENSSHAP00000005046_exon11 | scaffold236 | 5 |
| ENSSHAP00000016450_exon2 | scaffold28 | 1 |  | ENSSHAP00000005401_exon2 | scaffold85 | 5 |
| ENSSHAP00000016476_exon11 | scaffold77 | 1 |  | ENSSHAP00000005629_exon5 | scaffold85 | 5 |
| ENSSHAP00000016549_exon6 | scaffold155 | 1 |  | ENSSHAP00000005937_exon14 | scaffold85 | 5 |
| ENSSHAP00000016599_exon4 | scaffold28 | 1 |  | ENSSHAP00000005945_exon1 | scaffold236 | 5 |
| ENSSHAP00000016732_exon4 | scaffold28 | 1 |  | ENSSHAP00000006154_exon4 | scaffold180 | 5 |
| ENSSHAP00000016772_exon4 | scaffold28 | 1 |  | ENSSHAP00000006187_exon7 | scaffold85 | 5 |
| ENSSHAP00000016817_exon2 | scaffold155 | 1 |  | ENSSHAP00000006197_exon1 | scaffold236 | 5 |
| ENSSHAP00000016830_exon1 | scaffold77 | 1 |  | ENSSHAP00000006255_exon3 | scaffold85 | 5 |
| ENSSHAP00000016830_exon5 | scaffold77 | 1 |  | ENSSHAP00000006285_exon9 | scaffold180 | 5 |
| ENSSHAP00000016937_exon10 | scaffold28 | 1 |  | ENSSHAP00000006299_exon3 | scaffold85 | 5 |
| ENSSHAP00000016956_exon12 | scaffold28 | 1 |  | ENSSHAP00000006375_exon8 | scaffold180 | 5 |
| ENSSHAP00000017024_exon13 | scaffold155 | 1 |  | ENSSHAP00000006375_exon8 | scaffold180 | 5 |
| ENSSHAP00000017147_exon11 | scaffold77 | 1 |  | ENSSHAP00000006436_exon26 | scaffold180 | 5 |
| ENSSHAP00000017147_exon2 | scaffold77 | 1 |  | ENSSHAP00000006440_exon4 | scaffold236 | 5 |
| ENSSHAP00000017218_exon1 | scaffold155 | 1 |  | ENSSHAP00000006466_exon5 | scaffold236 | 5 |
| ENSSHAP00000017363_exon8 | scaffold28 | 1 |  | ENSSHAP00000006472_exon5 | scaffold236 | 5 |
| ENSSHAP00000017379_exon3 | scaffold155 | 1 |  | ENSSHAP00000006553_exon8 | scaffold180 | 5 |
| ENSSHAP00000017383_exon1 | scaffold28 | 1 |  | ENSSHAP00000006558_exon2 | scaffold236 | 5 |
| ENSSHAP00000017386_exon1 | scaffold155 | 1 |  | ENSSHAP00000006890_exon17 | scaffold180 | 5 |
| ENSSHAP00000017400_exon2 | scaffold155 | 1 |  | ENSSHAP00000007112_exon4 | scaffold85 | 5 |
| ENSSHAP00000017424_exon2 | scaffold155 | 1 |  | ENSSHAP00000007182_exon4 | scaffold236 | 5 |
| ENSSHAP00000017501_exon3 | scaffold28 | 1 |  | ENSSHAP00000007365_exon1 | scaffold85 | 5 |
| ENSSHAP00000017586_exon7 | scaffold28 | 1 |  | ENSSHAP00000007577_exon22 | scaffold236 | 5 |
| ENSSHAP00000017616_exon3 | scaffold155 | 1 |  | ENSSHAP00000007596_exon1 | scaffold236 | 5 |
| ENSSHAP00000017655_exon4 | scaffold28 | 1 |  | ENSSHAP00000007635_exon6 | scaffold85 | 5 |
| ENSSHAP00000017688_exon8 | scaffold28 | 1 |  | ENSSHAP00000007949_exon3 | scaffold85 | 5 |
| ENSSHAP00000017761_exon4 | scaffold28 | 1 |  | ENSSHAP00000007983_exon2 | scaffold85 | 5 |
| ENSSHAP00000018109_exon2 | scaffold77 | 1 |  | ENSSHAP00000008113_exon15 | scaffold236 | 5 |
| ENSSHAP00000018252_exon1 | scaffold155 | 1 |  | ENSSHAP00000008159_exon10 | scaffold236 | 5 |
| ENSSHAP00000018252_exon16 | scaffold155 | 1 |  | ENSSHAP00000008988_exon20 | scaffold236 | 5 |
| ENSSHAP00000018350_exon24 | scaffold155 | 1 |  | ENSSHAP00000009011_exon13 | scaffold180 | 5 |
| ENSSHAP00000018393_exon3 | scaffold155 | 1 |  | ENSSHAP00000009257_exon6 | scaffold236 | 5 |
| ENSSHAP00000018427_exon9 | scaffold155 | 1 |  | ENSSHAP00000009280_exon12 | scaffold85 | 5 |
| ENSSHAP00000018557_exon12 | scaffold155 | 1 |  | ENSSHAP00000009398_exon2 | scaffold180 | 5 |
| ENSSHAP00000018602_exon13 | scaffold28 | 1 |  | ENSSHAP00000009688_exon1 | scaffold236 | 5 |
| ENSSHAP00000018631_exon2 | scaffold77 | 1 |  | ENSSHAP00000009882_exon2 | scaffold85 | 5 |
| ENSSHAP00000018697_exon2 | scaffold77 | 1 |  | ENSSHAP00000010130_exon13 | scaffold85 | 5 |
| ENSSHAP00000018727_exon10 | scaffold77 | 1 |  | ENSSHAP00000010438_exon2 | scaffold236 | 5 |
| ENSSHAP00000018732_exon2 | scaffold28 | 1 |  | ENSSHAP00000010711_exon7 | scaffold236 | 5 |
| ENSSHAP00000019050_exon14 | scaffold28 | 1 |  | ENSSHAP00000011027_exon10 | scaffold85 | 5 |
| ENSSHAP00000019075_exon2 | scaffold77 | 1 |  | ENSSHAP00000011433_exon2 | scaffold85 | 5 |
| ENSSHAP00000019141_exon9 | scaffold155 | 1 |  | ENSSHAP00000011700_exon3 | scaffold180 | 5 |
| ENSSHAP00000019497_exon10 | scaffold77 | 1 |  | ENSSHAP00000011968_exon2 | scaffold180 | 5 |
| ENSSHAP00000019565_exon4 | scaffold155 | 1 |  | ENSSHAP00000011968_exon3 | scaffold180 | 5 |
| ENSSHAP00000019600_exon21 | scaffold155 | 1 |  | ENSSHAP00000012169_exon21 | scaffold180 | 5 |
| ENSSHAP00000019683_exon3 | scaffold28 | 1 |  | ENSSHAP00000012299_exon1 | scaffold180 | 5 |
| ENSSHAP00000019718_exon1 | scaffold155 | 1 |  | ENSSHAP00000012452_exon1 | scaffold85 | 5 |
| ENSSHAP00000019723_exon33 | scaffold28 | 1 |  | ENSSHAP00000012732_exon8 | scaffold85 | 5 |
| ENSSHAP00000000194_exon1 | scaffold65 | 2 |  | ENSSHAP00000012958_exon4 | scaffold85 | 5 |
| ENSSHAP00000000194_exon1 | scaffold65 | 2 |  | ENSSHAP00000013026_exon5 | scaffold85 | 5 |
| ENSSHAP00000000503_exon5 | scaffold121 | 2 |  | ENSSHAP00000013093_exon3 | scaffold85 | 5 |
| ENSSHAP00000000898_exon6 | scaffold65 | 2 |  | ENSSHAP00000013128_exon7 | scaffold85 | 5 |
| ENSSHAP00000001127_exon26 | scaffold121 | 2 |  | ENSSHAP00000013160_exon8 | scaffold180 | 5 |
| ENSSHAP00000001505_exon27 | scaffold121 | 2 |  | ENSSHAP00000013170_exon2 | scaffold180 | 5 |
| ENSSHAP00000001680_exon14 | scaffold65 | 2 |  | ENSSHAP00000013706_exon5 | scaffold85 | 5 |
| ENSSHAP00000001815_exon2 | scaffold121 | 2 |  | ENSSHAP00000013904_exon3 | scaffold180 | 5 |
| ENSSHAP00000002047_exon4 | scaffold121 | 2 |  | ENSSHAP00000014040_exon21 | scaffold85 | 5 |
| ENSSHAP00000002322_exon2 | scaffold65 | 2 |  | ENSSHAP00000014719_exon1 | scaffold180 | 5 |
| ENSSHAP00000002386_exon2 | scaffold121 | 2 |  | ENSSHAP00000015270_exon3 | scaffold180 | 5 |
| ENSSHAP00000002647_exon10 | scaffold121 | 2 |  | ENSSHAP00000015495_exon2 | scaffold180 | 5 |
| ENSSHAP00000002649_exon8 | scaffold65 | 2 |  | ENSSHAP00000015715_exon1 | scaffold85 | 5 |
| ENSSHAP00000003094_exon2 | scaffold65 | 2 |  | ENSSHAP00000015803_exon7 | scaffold180 | 5 |
| ENSSHAP00000003446_exon10 | scaffold65 | 2 |  | ENSSHAP00000016005_exon2 | scaffold180 | 5 |
| ENSSHAP00000003749_exon1 | scaffold65 | 2 |  | ENSSHAP00000016229_exon1 | scaffold180 | 5 |
| ENSSHAP00000005018_exon2 | scaffold121 | 2 |  | ENSSHAP00000016319_exon25 | scaffold180 | 5 |
| ENSSHAP00000005359_exon1 | scaffold121 | 2 |  | ENSSHAP00000016514_exon4 | scaffold85 | 5 |
| ENSSHAP00000005574_exon1 | scaffold65 | 2 |  | ENSSHAP00000016679_exon1 | scaffold85 | 5 |
| ENSSHAP00000005655_exon10 | scaffold65 | 2 |  | ENSSHAP00000016806_exon1 | scaffold236 | 5 |
| ENSSHAP00000006755_exon2 | scaffold65 | 2 |  | ENSSHAP00000016806_exon1 | scaffold236 | 5 |
| ENSSHAP00000006896_exon14 | scaffold121 | 2 |  | ENSSHAP00000016806_exon1 | scaffold236 | 5 |
| ENSSHAP00000007028_exon3 | scaffold121 | 2 |  | ENSSHAP00000017531_exon3 | scaffold180 | 5 |
| ENSSHAP00000007483_exon13 | scaffold121 | 2 |  | ENSSHAP00000018578_exon2 | scaffold85 | 5 |
| ENSSHAP00000007510_exon5 | scaffold65 | 2 |  | ENSSHAP00000018674_exon16 | scaffold85 | 5 |
| ENSSHAP00000007642_exon9 | scaffold121 | 2 |  | ENSSHAP00000019011_exon5 | scaffold85 | 5 |
| ENSSHAP00000008058_exon7 | scaffold65 | 2 |  | ENSSHAP00000019759_exon7 | scaffold180 | 5 |
| ENSSHAP00000008689_exon5 | scaffold65 | 2 |  | ENSSHAP00000002042_exon5 | scaffold113 | 6 |
| ENSSHAP00000008920_exon1 | scaffold65 | 2 |  | ENSSHAP00000002042_exon5 | scaffold113 | 6 |
| ENSSHAP00000009083_exon16 | scaffold121 | 2 |  | ENSSHAP00000002042_exon5 | scaffold113 | 6 |
| ENSSHAP00000009523_exon1 | scaffold121 | 2 |  | ENSSHAP00000002042_exon5 | scaffold113 | 6 |
| ENSSHAP00000009550_exon4 | scaffold65 | 2 |  | ENSSHAP00000002042_exon5 | scaffold113 | 6 |
| ENSSHAP00000009979_exon1 | scaffold121 | 2 |  | ENSSHAP00000002042_exon5 | scaffold113 | 6 |
| ENSSHAP00000010278_exon4 | scaffold65 | 2 |  | ENSSHAP00000002144_exon1 | scaffold113 | 6 |
| ENSSHAP00000010818_exon1 | scaffold65 | 2 |  | ENSSHAP00000004706_exon2 | scaffold113 | 6 |
| ENSSHAP00000011571_exon1 | scaffold121 | 2 |  | ENSSHAP00000004940_exon9 | scaffold113 | 6 |
| ENSSHAP00000011571_exon1 | scaffold121 | 2 |  | ENSSHAP00000011920_exon32 | scaffold113 | 6 |
| ENSSHAP00000011721_exon1 | scaffold65 | 2 |  | ENSSHAP00000013100_exon1 | scaffold113 | 6 |
| ENSSHAP00000011721_exon2 | scaffold65 | 2 |  | ENSSHAP00000014551_exon8 | scaffold113 | 6 |
| ENSSHAP00000011756_exon4 | scaffold65 | 2 |  | ENSSHAP00000016585_exon10 | scaffold113 | 6 |
| ENSSHAP00000012005_exon7 | scaffold65 | 2 |  | ENSSHAP00000019188_exon2 | scaffold113 | 6 |
| ENSSHAP00000012464_exon12 | scaffold65 | 2 |  | ENSSHAP00000000285_exon23 | scaffold9 | 7 |
| ENSSHAP00000012464_exon12 | scaffold65 | 2 |  | ENSSHAP00000000285_exon26 | scaffold9 | 7 |
| ENSSHAP00000012610_exon26 | scaffold65 | 2 |  | ENSSHAP00000000566_exon21 | scaffold131 | 7 |
| ENSSHAP00000012622_exon2 | scaffold65 | 2 |  | ENSSHAP00000000791_exon2 | scaffold3 | 7 |
| ENSSHAP00000012681_exon2 | scaffold121 | 2 |  | ENSSHAP00000000818_exon2 | scaffold131 | 7 |
| ENSSHAP00000012682_exon4 | scaffold121 | 2 |  | ENSSHAP00000000852_exon12 | scaffold131 | 7 |
| ENSSHAP00000013389_exon15 | scaffold65 | 2 |  | ENSSHAP00000000852_exon16 | scaffold131 | 7 |
| ENSSHAP00000013389_exon15 | scaffold65 | 2 |  | ENSSHAP00000001927_exon1 | scaffold131 | 7 |
| ENSSHAP00000013418_exon9 | scaffold65 | 2 |  | ENSSHAP00000002011_exon4 | scaffold3 | 7 |
| ENSSHAP00000013808_exon14 | scaffold65 | 2 |  | ENSSHAP00000002011_exon4 | scaffold3 | 7 |
| ENSSHAP00000014153_exon5 | scaffold121 | 2 |  | ENSSHAP00000002155_exon2 | scaffold9 | 7 |
| ENSSHAP00000014153_exon5 | scaffold121 | 2 |  | ENSSHAP00000002286_exon9 | scaffold9 | 7 |
| ENSSHAP00000014153_exon5 | scaffold121 | 2 |  | ENSSHAP00000002317_exon2 | scaffold3 | 7 |
| ENSSHAP00000014164_exon1 | scaffold121 | 2 |  | ENSSHAP00000002364_exon3 | scaffold3 | 7 |
| ENSSHAP00000014171_exon10 | scaffold65 | 2 |  | ENSSHAP00000002386_exon2 | scaffold9 | 7 |
| ENSSHAP00000014323_exon1 | scaffold121 | 2 |  | ENSSHAP00000002411_exon5 | scaffold9 | 7 |
| ENSSHAP00000014963_exon1 | scaffold65 | 2 |  | ENSSHAP00000002432_exon3 | scaffold131 | 7 |
| ENSSHAP00000015534_exon5 | scaffold65 | 2 |  | ENSSHAP00000002544_exon6 | scaffold131 | 7 |
| ENSSHAP00000015626_exon7 | scaffold65 | 2 |  | ENSSHAP00000002699_exon1 | scaffold9 | 7 |
| ENSSHAP00000015675_exon1 | scaffold65 | 2 |  | ENSSHAP00000002699_exon1 | scaffold9 | 7 |
| ENSSHAP00000015698_exon7 | scaffold65 | 2 |  | ENSSHAP00000002736_exon4 | scaffold3 | 7 |
| ENSSHAP00000016009_exon4 | scaffold121 | 2 |  | ENSSHAP00000002881_exon6 | scaffold3 | 7 |
| ENSSHAP00000016068_exon1 | scaffold65 | 2 |  | ENSSHAP00000003151_exon2 | scaffold9 | 7 |
| ENSSHAP00000016115_exon7 | scaffold121 | 2 |  | ENSSHAP00000003156_exon3 | scaffold131 | 7 |
| ENSSHAP00000016149_exon6 | scaffold65 | 2 |  | ENSSHAP00000003181_exon6 | scaffold131 | 7 |
| ENSSHAP00000016748_exon12 | scaffold65 | 2 |  | ENSSHAP00000003547_exon1 | scaffold131 | 7 |
| ENSSHAP00000016748_exon43 | scaffold65 | 2 |  | ENSSHAP00000003676_exon4 | scaffold131 | 7 |
| ENSSHAP00000016865_exon1 | scaffold121 | 2 |  | ENSSHAP00000004082_exon10 | scaffold9 | 7 |
| ENSSHAP00000016971_exon1 | scaffold65 | 2 |  | ENSSHAP00000004136_exon1 | scaffold9 | 7 |
| ENSSHAP00000017081_exon9 | scaffold121 | 2 |  | ENSSHAP00000004142_exon108 | scaffold131 | 7 |
| ENSSHAP00000017164_exon1 | scaffold65 | 2 |  | ENSSHAP00000004142_exon28 | scaffold131 | 7 |
| ENSSHAP00000017329_exon5 | scaffold65 | 2 |  | ENSSHAP00000004142_exon41 | scaffold131 | 7 |
| ENSSHAP00000017329_exon9 | scaffold65 | 2 |  | ENSSHAP00000004142_exon49 | scaffold131 | 7 |
| ENSSHAP00000017369_exon6 | scaffold121 | 2 |  | ENSSHAP00000004371_exon8 | scaffold9 | 7 |
| ENSSHAP00000017373_exon5 | scaffold65 | 2 |  | ENSSHAP00000004789_exon3 | scaffold3 | 7 |
| ENSSHAP00000017520_exon3 | scaffold121 | 2 |  | ENSSHAP00000004999_exon2 | scaffold9 | 7 |
| ENSSHAP00000017736_exon3 | scaffold121 | 2 |  | ENSSHAP00000005034_exon8 | scaffold9 | 7 |
| ENSSHAP00000018196_exon8 | scaffold65 | 2 |  | ENSSHAP00000005077_exon1 | scaffold3 | 7 |
| ENSSHAP00000018287_exon10 | scaffold65 | 2 |  | ENSSHAP00000005161_exon1 | scaffold9 | 7 |
| ENSSHAP00000018486_exon2 | scaffold121 | 2 |  | ENSSHAP00000005166_exon5 | scaffold3 | 7 |
| ENSSHAP00000018991_exon10 | scaffold65 | 2 |  | ENSSHAP00000005249_exon5 | scaffold9 | 7 |
| ENSSHAP00000018991_exon26 | scaffold65 | 2 |  | ENSSHAP00000005607_exon6 | scaffold9 | 7 |
| ENSSHAP00000018991_exon4 | scaffold65 | 2 |  | ENSSHAP00000005661_exon3 | scaffold9 | 7 |
| ENSSHAP00000018991_exon47 | scaffold65 | 2 |  | ENSSHAP00000005746_exon2 | scaffold3 | 7 |
| ENSSHAP00000019425_exon2 | scaffold121 | 2 |  | ENSSHAP00000006276_exon4 | scaffold131 | 7 |
| ENSSHAP00000019511_exon2 | scaffold121 | 2 |  | ENSSHAP00000006276_exon4 | scaffold131 | 7 |
| ENSSHAP00000019737_exon3 | scaffold65 | 2 |  | ENSSHAP00000006281_exon3 | scaffold9 | 7 |
| ENSSHAP00000000662_exon6 | scaffold6 | 3 |  | ENSSHAP00000006388_exon1 | scaffold131 | 7 |
| ENSSHAP00000001583_exon15 | scaffold63 | 3 |  | ENSSHAP00000006388_exon19 | scaffold131 | 7 |
| ENSSHAP00000001705_exon8 | scaffold6 | 3 |  | ENSSHAP00000006388_exon5 | scaffold131 | 7 |
| ENSSHAP00000001747_exon8 | scaffold6 | 3 |  | ENSSHAP00000006831_exon1 | scaffold131 | 7 |
| ENSSHAP00000002386_exon2 | scaffold63 | 3 |  | ENSSHAP00000006831_exon1 | scaffold131 | 7 |
| ENSSHAP00000002391_exon5 | scaffold76 | 3 |  | ENSSHAP00000007061_exon14 | scaffold131 | 7 |
| ENSSHAP00000002493_exon5 | scaffold63 | 3 |  | ENSSHAP00000007072_exon2 | scaffold131 | 7 |
| ENSSHAP00000002516_exon20 | scaffold6 | 3 |  | ENSSHAP00000007195_exon13 | scaffold9 | 7 |
| ENSSHAP00000002637_exon11 | scaffold63 | 3 |  | ENSSHAP00000007195_exon21 | scaffold9 | 7 |
| ENSSHAP00000002745_exon34 | scaffold6 | 3 |  | ENSSHAP00000007230_exon1 | scaffold9 | 7 |
| ENSSHAP00000002872_exon14 | scaffold76 | 3 |  | ENSSHAP00000007415_exon10 | scaffold3 | 7 |
| ENSSHAP00000002958_exon2 | scaffold343 | 3 |  | ENSSHAP00000007536_exon13 | scaffold131 | 7 |
| ENSSHAP00000003109_exon2 | scaffold76 | 3 |  | ENSSHAP00000007670_exon10 | scaffold9 | 7 |
| ENSSHAP00000003224_exon17 | scaffold6 | 3 |  | ENSSHAP00000007722_exon3 | scaffold9 | 7 |
| ENSSHAP00000003275_exon3 | scaffold63 | 3 |  | ENSSHAP00000007842_exon2 | scaffold9 | 7 |
| ENSSHAP00000003275_exon3 | scaffold6 | 3 |  | ENSSHAP00000008129_exon19 | scaffold3 | 7 |
| ENSSHAP00000003369_exon8 | scaffold343 | 3 |  | ENSSHAP00000008668_exon6 | scaffold131 | 7 |
| ENSSHAP00000003451_exon4 | scaffold76 | 3 |  | ENSSHAP00000008683_exon3 | scaffold3 | 7 |
| ENSSHAP00000003589_exon1 | scaffold63 | 3 |  | ENSSHAP00000008855_exon4 | scaffold131 | 7 |
| ENSSHAP00000003636_exon1 | scaffold76 | 3 |  | ENSSHAP00000009278_exon6 | scaffold131 | 7 |
| ENSSHAP00000003757_exon5 | scaffold63 | 3 |  | ENSSHAP00000009824_exon9 | scaffold131 | 7 |
| ENSSHAP00000003860_exon3 | scaffold63 | 3 |  | ENSSHAP00000009920_exon2 | scaffold131 | 7 |
| ENSSHAP00000004366_exon14 | scaffold6 | 3 |  | ENSSHAP00000009974_exon4 | scaffold3 | 7 |
| ENSSHAP00000004487_exon6 | scaffold6 | 3 |  | ENSSHAP00000010345_exon36 | scaffold9 | 7 |
| ENSSHAP00000004661_exon12 | scaffold76 | 3 |  | ENSSHAP00000010398_exon5 | scaffold131 | 7 |
| ENSSHAP00000004885_exon3 | scaffold76 | 3 |  | ENSSHAP00000010539_exon1 | scaffold131 | 7 |
| ENSSHAP00000005716_exon2 | scaffold6 | 3 |  | ENSSHAP00000011483_exon6 | scaffold3 | 7 |
| ENSSHAP00000005808_exon6 | scaffold63 | 3 |  | ENSSHAP00000011726_exon1 | scaffold131 | 7 |
| ENSSHAP00000005859_exon1 | scaffold63 | 3 |  | ENSSHAP00000011764_exon5 | scaffold9 | 7 |
| ENSSHAP00000005893_exon23 | scaffold6 | 3 |  | ENSSHAP00000011846_exon1 | scaffold131 | 7 |
| ENSSHAP00000005893_exon39 | scaffold6 | 3 |  | ENSSHAP00000012612_exon13 | scaffold9 | 7 |
| ENSSHAP00000005916_exon4 | scaffold63 | 3 |  | ENSSHAP00000012723_exon11 | scaffold3 | 7 |
| ENSSHAP00000006281_exon3 | scaffold6 | 3 |  | ENSSHAP00000012724_exon8 | scaffold9 | 7 |
| ENSSHAP00000006281_exon3 | scaffold6 | 3 |  | ENSSHAP00000012834_exon6 | scaffold9 | 7 |
| ENSSHAP00000006379_exon1 | scaffold76 | 3 |  | ENSSHAP00000013555_exon2 | scaffold131 | 7 |
| ENSSHAP00000006756_exon19 | scaffold76 | 3 |  | ENSSHAP00000013619_exon2 | scaffold9 | 7 |
| ENSSHAP00000006815_exon2 | scaffold76 | 3 |  | ENSSHAP00000013621_exon1 | scaffold9 | 7 |
| ENSSHAP00000007011_exon5 | scaffold63 | 3 |  | ENSSHAP00000013712_exon5 | scaffold131 | 7 |
| ENSSHAP00000007015_exon13 | scaffold6 | 3 |  | ENSSHAP00000013827_exon4 | scaffold9 | 7 |
| ENSSHAP00000007270_exon1 | scaffold76 | 3 |  | ENSSHAP00000014138_exon4 | scaffold3 | 7 |
| ENSSHAP00000007497_exon1 | scaffold343 | 3 |  | ENSSHAP00000014188_exon12 | scaffold131 | 7 |
| ENSSHAP00000007540_exon6 | scaffold63 | 3 |  | ENSSHAP00000014232_exon1 | scaffold131 | 7 |
| ENSSHAP00000007677_exon6 | scaffold76 | 3 |  | ENSSHAP00000014395_exon5 | scaffold131 | 7 |
| ENSSHAP00000007778_exon8 | scaffold76 | 3 |  | ENSSHAP00000014543_exon3 | scaffold131 | 7 |
| ENSSHAP00000007797_exon7 | scaffold6 | 3 |  | ENSSHAP00000014670_exon15 | scaffold3 | 7 |
| ENSSHAP00000007810_exon1 | scaffold63 | 3 |  | ENSSHAP00000014727_exon22 | scaffold131 | 7 |
| ENSSHAP00000007949_exon3 | scaffold76 | 3 |  | ENSSHAP00000014727_exon35 | scaffold131 | 7 |
| ENSSHAP00000007960_exon1 | scaffold6 | 3 |  | ENSSHAP00000014963_exon1 | scaffold3 | 7 |
| ENSSHAP00000007996_exon9 | scaffold63 | 3 |  | ENSSHAP00000015536_exon1 | scaffold9 | 7 |
| ENSSHAP00000008183_exon11 | scaffold76 | 3 |  | ENSSHAP00000015594_exon24 | scaffold9 | 7 |
| ENSSHAP00000008206_exon6 | scaffold343 | 3 |  | ENSSHAP00000015713_exon17 | scaffold9 | 7 |
| ENSSHAP00000008309_exon2 | scaffold343 | 3 |  | ENSSHAP00000016048_exon11 | scaffold3 | 7 |
| ENSSHAP00000008333_exon16 | scaffold6 | 3 |  | ENSSHAP00000016048_exon6 | scaffold3 | 7 |
| ENSSHAP00000008496_exon13 | scaffold63 | 3 |  | ENSSHAP00000016050_exon2 | scaffold131 | 7 |
| ENSSHAP00000008496_exon13 | scaffold63 | 3 |  | ENSSHAP00000016081_exon2 | scaffold131 | 7 |
| ENSSHAP00000008772_exon2 | scaffold63 | 3 |  | ENSSHAP00000016492_exon10 | scaffold3 | 7 |
| ENSSHAP00000008920_exon1 | scaffold6 | 3 |  | ENSSHAP00000016654_exon7 | scaffold3 | 7 |
| ENSSHAP00000008937_exon3 | scaffold76 | 3 |  | ENSSHAP00000016661_exon2 | scaffold9 | 7 |
| ENSSHAP00000009004_exon3 | scaffold6 | 3 |  | ENSSHAP00000016740_exon14 | scaffold3 | 7 |
| ENSSHAP00000009029_exon9 | scaffold63 | 3 |  | ENSSHAP00000016799_exon8 | scaffold3 | 7 |
| ENSSHAP00000009182_exon14 | scaffold63 | 3 |  | ENSSHAP00000016942_exon20 | scaffold9 | 7 |
| ENSSHAP00000009198_exon9 | scaffold76 | 3 |  | ENSSHAP00000017168_exon2 | scaffold3 | 7 |
| ENSSHAP00000009202_exon2 | scaffold343 | 3 |  | ENSSHAP00000017259_exon2 | scaffold9 | 7 |
| ENSSHAP00000009295_exon10 | scaffold6 | 3 |  | ENSSHAP00000017259_exon2 | scaffold131 | 7 |
| ENSSHAP00000009307_exon10 | scaffold63 | 3 |  | ENSSHAP00000017522_exon18 | scaffold3 | 7 |
| ENSSHAP00000009352_exon3 | scaffold63 | 3 |  | ENSSHAP00000017691_exon7 | scaffold9 | 7 |
| ENSSHAP00000009352_exon3 | scaffold76 | 3 |  | ENSSHAP00000017691_exon7 | scaffold131 | 7 |
| ENSSHAP00000009523_exon1 | scaffold343 | 3 |  | ENSSHAP00000017855_exon16 | scaffold9 | 7 |
| ENSSHAP00000009784_exon8 | scaffold6 | 3 |  | ENSSHAP00000018320_exon11 | scaffold3 | 7 |
| ENSSHAP00000009860_exon22 | scaffold6 | 3 |  | ENSSHAP00000018320_exon74 | scaffold3 | 7 |
| ENSSHAP00000009895_exon19 | scaffold63 | 3 |  | ENSSHAP00000018567_exon1 | scaffold3 | 7 |
| ENSSHAP00000010026_exon4 | scaffold63 | 3 |  | ENSSHAP00000018567_exon1 | scaffold3 | 7 |
| ENSSHAP00000010184_exon1 | scaffold63 | 3 |  | ENSSHAP00000018886_exon2 | scaffold131 | 7 |
| ENSSHAP00000010197_exon5 | scaffold63 | 3 |  | ENSSHAP00000019010_exon7 | scaffold9 | 7 |
| ENSSHAP00000010203_exon13 | scaffold76 | 3 |  | ENSSHAP00000019268_exon17 | scaffold9 | 7 |
| ENSSHAP00000010205_exon15 | scaffold76 | 3 |  | ENSSHAP00000019268_exon28 | scaffold9 | 7 |
| ENSSHAP00000010221_exon1 | scaffold343 | 3 |  | ENSSHAP00000019268_exon28 | scaffold9 | 7 |
| ENSSHAP00000010246_exon8 | scaffold76 | 3 |  | ENSSHAP00000019799_exon31 | scaffold3 | 7 |
| ENSSHAP00000010255_exon7 | scaffold63 | 3 |  | ENSSHAP00000001780_exon20 | scaffold42 | 8 |
| ENSSHAP00000010318_exon4 | scaffold63 | 3 |  | ENSSHAP00000001780_exon5 | scaffold42 | 8 |
| ENSSHAP00000010525_exon11 | scaffold343 | 3 |  | ENSSHAP00000003094_exon2 | scaffold42 | 8 |
| ENSSHAP00000010555_exon3 | scaffold63 | 3 |  | ENSSHAP00000003846_exon2 | scaffold42 | 8 |
| ENSSHAP00000010584_exon7 | scaffold63 | 3 |  | ENSSHAP00000003867_exon34 | scaffold42 | 8 |
| ENSSHAP00000010602_exon3 | scaffold63 | 3 |  | ENSSHAP00000007797_exon7 | scaffold42 | 8 |
| ENSSHAP00000010602_exon3 | scaffold63 | 3 |  | ENSSHAP00000008225_exon25 | scaffold42 | 8 |
| ENSSHAP00000010613_exon7 | scaffold343 | 3 |  | ENSSHAP00000008842_exon1 | scaffold42 | 8 |
| ENSSHAP00000010749_exon6 | scaffold343 | 3 |  | ENSSHAP00000008842_exon17 | scaffold42 | 8 |
| ENSSHAP00000011016_exon11 | scaffold76 | 3 |  | ENSSHAP00000009936_exon12 | scaffold42 | 8 |
| ENSSHAP00000011032_exon7 | scaffold63 | 3 |  | ENSSHAP00000010684_exon4 | scaffold42 | 8 |
| ENSSHAP00000011334_exon2 | scaffold6 | 3 |  | ENSSHAP00000011605_exon3 | scaffold42 | 8 |
| ENSSHAP00000011350_exon12 | scaffold343 | 3 |  | ENSSHAP00000012235_exon1 | scaffold42 | 8 |
| ENSSHAP00000011452_exon17 | scaffold76 | 3 |  | ENSSHAP00000012343_exon6 | scaffold42 | 8 |
| ENSSHAP00000011545_exon4 | scaffold343 | 3 |  | ENSSHAP00000012517_exon6 | scaffold42 | 8 |
| ENSSHAP00000011571_exon1 | scaffold343 | 3 |  | ENSSHAP00000012894_exon10 | scaffold42 | 8 |
| ENSSHAP00000011584_exon4 | scaffold6 | 3 |  | ENSSHAP00000013349_exon2 | scaffold42 | 8 |
| ENSSHAP00000011584_exon4 | scaffold6 | 3 |  | ENSSHAP00000013354_exon2 | scaffold42 | 8 |
| ENSSHAP00000011611_exon5 | scaffold6 | 3 |  | ENSSHAP00000013781_exon8 | scaffold42 | 8 |
| ENSSHAP00000011611_exon5 | scaffold6 | 3 |  | ENSSHAP00000014011_exon10 | scaffold42 | 8 |
| ENSSHAP00000011661_exon3 | scaffold76 | 3 |  | ENSSHAP00000014561_exon17 | scaffold42 | 8 |
| ENSSHAP00000011663_exon2 | scaffold76 | 3 |  | ENSSHAP00000014845_exon2 | scaffold42 | 8 |
| ENSSHAP00000011852_exon1 | scaffold76 | 3 |  | ENSSHAP00000015187_exon3 | scaffold42 | 8 |
| ENSSHAP00000011860_exon10 | scaffold76 | 3 |  | ENSSHAP00000017019_exon3 | scaffold42 | 8 |
| ENSSHAP00000011862_exon2 | scaffold63 | 3 |  | ENSSHAP00000017337_exon2 | scaffold42 | 8 |
| ENSSHAP00000011884_exon23 | scaffold343 | 3 |  | ENSSHAP00000017337_exon4 | scaffold42 | 8 |
| ENSSHAP00000011902_exon4 | scaffold63 | 3 |  | ENSSHAP00000018044_exon6 | scaffold42 | 8 |
| ENSSHAP00000012031_exon14 | scaffold76 | 3 |  | ENSSHAP00000018175_exon3 | scaffold42 | 8 |
| ENSSHAP00000012078_exon15 | scaffold343 | 3 |  | ENSSHAP00000018238_exon10 | scaffold42 | 8 |
| ENSSHAP00000012285_exon17 | scaffold6 | 3 |  | ENSSHAP00000018464_exon2 | scaffold42 | 8 |
| ENSSHAP00000012324_exon2 | scaffold76 | 3 |  | ENSSHAP00000018655_exon8 | scaffold42 | 8 |
| ENSSHAP00000012343_exon6 | scaffold63 | 3 |  | ENSSHAP00000018838_exon11 | scaffold42 | 8 |
| ENSSHAP00000012396_exon5 | scaffold6 | 3 |  | ENSSHAP00000019033_exon1 | scaffold42 | 8 |
| ENSSHAP00000012500_exon9 | scaffold6 | 3 |  | ENSSHAP00000019261_exon2 | scaffold42 | 8 |
| ENSSHAP00000012712_exon6 | scaffold76 | 3 |  | ENSSHAP00000019419_exon2 | scaffold42 | 8 |
| ENSSHAP00000012712_exon6 | scaffold76 | 3 |  | ENSSHAP00000019419_exon2 | scaffold42 | 8 |
| ENSSHAP00000013056_exon5 | scaffold63 | 3 |  | ENSSHAP00000019419_exon2 | scaffold42 | 8 |
| ENSSHAP00000013056_exon9 | scaffold63 | 3 |  | ENSSHAP00000019419_exon2 | scaffold42 | 8 |
| ENSSHAP00000013097_exon1 | scaffold76 | 3 |  | ENSSHAP00000019682_exon2 | scaffold42 | 8 |
| ENSSHAP00000013228_exon2 | scaffold76 | 3 |  | ENSSHAP00000019682_exon8 | scaffold42 | 8 |
| ENSSHAP00000013353_exon4 | scaffold76 | 3 |  | ENSSHAP00000019757_exon7 | scaffold42 | 8 |
| ENSSHAP00000013694_exon6 | scaffold343 | 3 |  | ENSSHAP00000004308_exon4 | scaffold479 | 9 |
| ENSSHAP00000013715_exon12 | scaffold6 | 3 |  | ENSSHAP00000007509_exon9 | scaffold479 | 9 |
| ENSSHAP00000013808_exon14 | scaffold6 | 3 |  | ENSSHAP00000007672_exon4 | scaffold479 | 9 |
| ENSSHAP00000013825_exon13 | scaffold343 | 3 |  | ENSSHAP00000007797_exon7 | scaffold479 | 9 |
| ENSSHAP00000013825_exon14 | scaffold343 | 3 |  | ENSSHAP00000011204_exon3 | scaffold479 | 9 |
| ENSSHAP00000014026_exon15 | scaffold343 | 3 |  | ENSSHAP00000013570_exon17 | scaffold479 | 9 |
| ENSSHAP00000014256_exon2 | scaffold6 | 3 |  | ENSSHAP00000013818_exon3 | scaffold479 | 9 |
| ENSSHAP00000014281_exon2 | scaffold76 | 3 |  | ENSSHAP00000018486_exon2 | scaffold479 | 9 |
| ENSSHAP00000014341_exon3 | scaffold76 | 3 |  | ENSSHAP00000018743_exon7 | scaffold479 | 9 |
| ENSSHAP00000014341_exon7 | scaffold76 | 3 |  | ENSSHAP00000000295_exon1 | scaffold123 | 10 |
| ENSSHAP00000014399_exon5 | scaffold76 | 3 |  | ENSSHAP00000002935_exon4 | scaffold123 | 10 |
| ENSSHAP00000014399_exon9 | scaffold76 | 3 |  | ENSSHAP00000002935_exon4 | scaffold123 | 10 |
| ENSSHAP00000014466_exon8 | scaffold63 | 3 |  | ENSSHAP00000003116_exon5 | scaffold123 | 10 |
| ENSSHAP00000014549_exon2 | scaffold343 | 3 |  | ENSSHAP00000004036_exon2 | scaffold123 | 10 |
| ENSSHAP00000014610_exon23 | scaffold343 | 3 |  | ENSSHAP00000004117_exon1 | scaffold123 | 10 |
| ENSSHAP00000014807_exon2 | scaffold343 | 3 |  | ENSSHAP00000004454_exon30 | scaffold123 | 10 |
| ENSSHAP00000014845_exon2 | scaffold6 | 3 |  | ENSSHAP00000004454_exon36 | scaffold123 | 10 |
| ENSSHAP00000015373_exon4 | scaffold76 | 3 |  | ENSSHAP00000005410_exon17 | scaffold123 | 10 |
| ENSSHAP00000015509_exon8 | scaffold343 | 3 |  | ENSSHAP00000005410_exon17 | scaffold123 | 10 |
| ENSSHAP00000015741_exon4 | scaffold343 | 3 |  | ENSSHAP00000005410_exon17 | scaffold123 | 10 |
| ENSSHAP00000015954_exon9 | scaffold6 | 3 |  | ENSSHAP00000006214_exon6 | scaffold123 | 10 |
| ENSSHAP00000016164_exon28 | scaffold76 | 3 |  | ENSSHAP00000008134_exon1 | scaffold123 | 10 |
| ENSSHAP00000016281_exon13 | scaffold76 | 3 |  | ENSSHAP00000008645_exon48 | scaffold123 | 10 |
| ENSSHAP00000016746_exon22 | scaffold63 | 3 |  | ENSSHAP00000009017_exon2 | scaffold123 | 10 |
| ENSSHAP00000016886_exon4 | scaffold76 | 3 |  | ENSSHAP00000009352_exon3 | scaffold123 | 10 |
| ENSSHAP00000016919_exon4 | scaffold343 | 3 |  | ENSSHAP00000010371_exon9 | scaffold123 | 10 |
| ENSSHAP00000016919_exon4 | scaffold343 | 3 |  | ENSSHAP00000010519_exon6 | scaffold123 | 10 |
| ENSSHAP00000017397_exon3 | scaffold6 | 3 |  | ENSSHAP00000010922_exon5 | scaffold123 | 10 |
| ENSSHAP00000017397_exon8 | scaffold6 | 3 |  | ENSSHAP00000011913_exon17 | scaffold123 | 10 |
| ENSSHAP00000017880_exon19 | scaffold343 | 3 |  | ENSSHAP00000011915_exon4 | scaffold123 | 10 |
| ENSSHAP00000018005_exon1 | scaffold63 | 3 |  | ENSSHAP00000012255_exon7 | scaffold123 | 10 |
| ENSSHAP00000018276_exon5 | scaffold343 | 3 |  | ENSSHAP00000012256_exon5 | scaffold123 | 10 |
| ENSSHAP00000018581_exon8 | scaffold343 | 3 |  | ENSSHAP00000012544_exon7 | scaffold123 | 10 |
| ENSSHAP00000018581_exon8 | scaffold343 | 3 |  | ENSSHAP00000013924_exon2 | scaffold123 | 10 |
| ENSSHAP00000018765_exon3 | scaffold343 | 3 |  | ENSSHAP00000014749_exon1 | scaffold123 | 10 |
| ENSSHAP00000019095_exon3 | scaffold343 | 3 |  | ENSSHAP00000015790_exon1 | scaffold123 | 10 |
| ENSSHAP00000019225_exon3 | scaffold343 | 3 |  | ENSSHAP00000016296_exon2 | scaffold123 | 10 |
| ENSSHAP00000019311_exon5 | scaffold6 | 3 |  | ENSSHAP00000017504_exon11 | scaffold123 | 10 |
| ENSSHAP00000019384_exon19 | scaffold343 | 3 |  | ENSSHAP00000017504_exon6 | scaffold123 | 10 |
| ENSSHAP00000019449_exon26 | scaffold63 | 3 |  | ENSSHAP00000018633_exon3 | scaffold123 | 10 |
| ENSSHAP00000019449_exon38 | scaffold63 | 3 |  | ENSSHAP00000019159_exon2 | scaffold123 | 10 |
| ENSSHAP00000019594_exon2 | scaffold343 | 3 |  | ENSSHAP00000000168_exon3 | scaffold183 | X |
| ENSSHAP00000019672_exon9 | scaffold76 | 3 |  | ENSSHAP00000000646_exon1 | scaffold183 | X |
| ENSSHAP00000019718_exon1 | scaffold6 | 3 |  | ENSSHAP00000001052_exon6 | scaffold183 | X |
| ENSSHAP00000000070_exon7 | scaffold33 | 4 |  | ENSSHAP00000001182_exon20 | scaffold876 | X |
| ENSSHAP00000000071_exon7 | scaffold33 | 4 |  | ENSSHAP00000001182_exon20 | scaffold876 | X |
| ENSSHAP00000000334_exon17 | scaffold33 | 4 |  | ENSSHAP00000001182_exon20 | scaffold876 | X |
| ENSSHAP00000001001_exon1 | scaffold33 | 4 |  | ENSSHAP00000001230_exon35 | scaffold183 | X |
| ENSSHAP00000001016_exon1 | scaffold33 | 4 |  | ENSSHAP00000001230_exon4 | scaffold183 | X |
| ENSSHAP00000001260_exon4 | scaffold33 | 4 |  | ENSSHAP00000001230_exon66 | scaffold183 | X |
| ENSSHAP00000001842_exon3 | scaffold351 | 4 |  | ENSSHAP00000002198_exon1 | scaffold183 | X |
| ENSSHAP00000001866_exon12 | scaffold33 | 4 |  | ENSSHAP00000002566_exon1 | scaffold876 | X |
| ENSSHAP00000001893_exon3 | scaffold27 | 4 |  | ENSSHAP00000002566_exon1 | scaffold876 | X |
| ENSSHAP00000001924_exon18 | scaffold351 | 4 |  | ENSSHAP00000002715_exon5 | scaffold183 | X |
| ENSSHAP00000002386_exon2 | scaffold27 | 4 |  | ENSSHAP00000003507_exon14 | scaffold183 | X |
| ENSSHAP00000002790_exon1 | scaffold33 | 4 |  | ENSSHAP00000004242_exon1 | scaffold183 | X |
| ENSSHAP00000002790_exon1 | scaffold33 | 4 |  | ENSSHAP00000004381_exon1 | scaffold876 | X |
| ENSSHAP00000002790_exon1 | scaffold33 | 4 |  | ENSSHAP00000004788_exon2 | scaffold183 | X |
| ENSSHAP00000002790_exon1 | scaffold33 | 4 |  | ENSSHAP00000006787_exon3 | scaffold183 | X |
| ENSSHAP00000002938_exon22 | scaffold27 | 4 |  | ENSSHAP00000007576_exon3 | scaffold977 | X |
| ENSSHAP00000003134_exon2 | scaffold351 | 4 |  | ENSSHAP00000008620_exon11 | scaffold183 | X |
| ENSSHAP00000003373_exon5 | scaffold33 | 4 |  | ENSSHAP00000008837_exon9 | scaffold977 | X |
| ENSSHAP00000003414_exon11 | scaffold27 | 4 |  | ENSSHAP00000009059_exon6 | scaffold977 | X |
| ENSSHAP00000003523_exon40 | scaffold33 | 4 |  | ENSSHAP00000009128_exon5 | scaffold183 | X |
| ENSSHAP00000003523_exon50 | scaffold33 | 4 |  | ENSSHAP00000010957_exon2 | scaffold183 | X |
| ENSSHAP00000004379_exon4 | scaffold33 | 4 |  | ENSSHAP00000011347_exon25 | scaffold183 | X |
| ENSSHAP00000004612_exon1 | scaffold351 | 4 |  | ENSSHAP00000011879_exon3 | scaffold183 | X |
| ENSSHAP00000004731_exon2 | scaffold27 | 4 |  | ENSSHAP00000012065_exon22 | scaffold183 | X |
| ENSSHAP00000004740_exon2 | scaffold33 | 4 |  | ENSSHAP00000012065_exon4 | scaffold183 | X |
| ENSSHAP00000004827_exon7 | scaffold33 | 4 |  | ENSSHAP00000013324_exon7 | scaffold876 | X |
| ENSSHAP00000004955_exon15 | scaffold351 | 4 |  | ENSSHAP00000016945_exon2 | scaffold977 | X |
| ENSSHAP00000004957_exon4 | scaffold27 | 4 |  | ENSSHAP00000019101_exon1 | scaffold876 | X |
| ENSSHAP00000005176_exon1 | scaffold351 | 4 |  | ENSSHAP00000019101_exon1 | scaffold876 | X |
| ENSSHAP00000005269_exon2 | scaffold27 | 4 |  | ENSSHAP00000019101_exon1 | scaffold876 | X |
| ENSSHAP00000005388_exon2 | scaffold351 | 4 |  | ENSSHAP00000019101_exon1 | scaffold876 | X |
| ENSSHAP00000005527_exon1 | scaffold351 | 4 |  | ENSSHAP00000019101_exon1 | scaffold876 | X |
| ENSSHAP00000005756_exon1 | scaffold33 | 4 |  | ENSSHAP00000019101_exon1 | scaffold876 | X |
