# Supplementary material for: Chromosomal Speciation in the Genomics Era: Disentangling Phylogenetic Evolution of Rock-wallabies
Source: Front Genet. 2017 Feb 10;8:10. doi: 10.3389/fgene.2017.00010 (PMC5301020; doi:10.3389/fgene.2017.00010)
Supplement: Supplementary file 4 [file Table_4.docx]

**Supplementary Table 4** Monophyly counts of sampled individuals in a taxon (species/sub-species/race) of *Petrogale*. This represents the number of times two individuals group to form a monophyletic lineage across the 100 bootstrap trees of the concatenated nuclear data, including all data, the X chromosome loci, non-rearranged (2,4,7,8) autosomal loci and the rearranged (5,6,9,10) loci. These counts were conducted for the *lateralis* and *penicillata* chromosomal groups only.

| ***penicillata* group** | **ALL** | **X** | **Non-rearranged** | **Rearranged** |
| --- | --- | --- | --- | --- |
| *assimilis* | 74 | 8 | 40 | 0 |
| *coenensis* | 92 | 100 | 82 | 71 |
| *godmani* | 97 | 74 | 9 | 90 |
| *herberti* | 70 | 99 | 7 | 90 |
| *inornata* | 100 | 99 | 58 | 50 |
| *mareeba* | 94 | 28 | 0 | 12 |
| *pencillata* | 100 | 96 | 100 | 94 |
| *sharmani* | 100 | 98 | 94 | 1 |
| **Average** | 90.875 | 75.25 | 48.75 | 51 |

| ***lateralis* group** | **ALL** | **X** | **Non-rearranged** | **Rearranged** |
| --- | --- | --- | --- | --- |
| *lateralis lateralis* | 15 | 7 | 2 | 3 |
| *lateralis pearsoni* | 100 | 70 | 100 | 76 |
| *lateralis* WK race | 56 | 9 | 1 | 7 |
| *lateralis* MR race | 100 | 100 | 15 | 2 |
| *lateralis hacketti* | 100 | 31 | 66 | 70 |
| **Average** | 74.2 | 43.4 | 36.8 | 31.6 |
