# Supplementary material for: Chromosomal Speciation in the Genomics Era: Disentangling Phylogenetic Evolution of Rock-wallabies
Source: Front Genet. 2017 Feb 10;8:10. doi: 10.3389/fgene.2017.00010 (PMC5301020; doi:10.3389/fgene.2017.00010)
Supplement: Supplementary file 5 [file Table_5.docx]

**Supplementary Table 5** Maximum Likelihood phylogenetic network results from PhyloNet analyses exploring reticulation in the evolutionary history of *Petrogale*. For each chromosomal group two independent runs were conducted with a single individual from each taxon in each analysis. Results include the log likelihood scores (lnL) for each model with reticulation from 0-3, and the AIC scores (2k – 2lnL; where k = free parameters). The lowest AIC score was chosen as the best model given the data and is highlighted for each analysis in bold.

| *penicillata* (a) |  |  |  | *penicillata* (b) |  |  |
| --- | --- | --- | --- | --- | --- | --- |
| **Reticulations** | **lnL** | **AIC** |  | **Reticulations** | **lnL** | **AIC** |
| 0 | -2834.733009 | 5677.466019 |  | 0 | -2619.024137 | 5246.048274 |
| **1** | **-2825.3457** | **5662.691399** |  | **1** | **-2611.390036** | **5234.780072** |
| 2 | -2825.344954 | 5666.689908 |  | 2 | -2613.673227 | 5243.346455 |
| 3 | -2826.334241 | 5672.668482 |  | 3 | -2612.306308 | 5244.612615 |
|  |  |  |  |  |  |  |
| *lateralis* (a) |  |  |  | *lateralis* (b) |  |  |
| **Reticulations** | **lnL** | **AIC** |  | **Reticulations** | **lnL** | **AIC** |
| 0 | -3005.235136 | 6018.470273 |  | 0 | -2874.531916 | 5757.063831 |
| **1** | **-2991.516327** | **5995.032654** |  | **1** | **-2863.410336** | **5738.820673** |
| 2 | -2992.357334 | 6000.714667 |  | 2 | -2863.396047 | 5742.792094 |
| 3 | -2991.271379 | 6002.542758 |  | 3 | -2863.22837 | 5746.45674 |
|  |  |  |  |  |  |  |
| *brachyotis* (a) |  |  |  | *brachyotis* (b) |  |  |
| **Reticulations** | **lnL** | AIC |  | **Reticulations** | **lnL** | **AIC** |
| 0 | -7470.877476 | 14949.75495 |  | 0 | -7328.128348 | 14664.2567 |
| 1 | -7374.738781 | 14761.47756 |  | 1 | -7256.99101 | 14525.98202 |
| 2 | -7300.181498 | 14616.363 |  | **2** | **-7171.572325** | **14359.14465** |
| **3** | **-7295.169035** | **14610.33807** |  | 3 | -7170.643981 | 14361.28796 |
