# Supplementary material for: Chromosomal Speciation in the Genomics Era: Disentangling Phylogenetic Evolution of Rock-wallabies
Source: Front Genet. 2017 Feb 10;8:10. doi: 10.3389/fgene.2017.00010 (PMC5301020; doi:10.3389/fgene.2017.00010)
Supplement: Supplementary file 6 [file Data_Sheet_1.docx]

**Supplementary - Materials and methods**

*Sampling and extraction*

We sampled individual representatives from 24 of the 25 chromosomal races/sub-species/species within the genus *Petrogale* (see in Supp. Table 1; Fig. 1). Most samples were obtained from the Australian Museum Research Institute’s tissue collection, as well at the Australian Biodiversity Tissue Collection from the South Australian Museum. We included two individuals for each taxon except for *P. concinna canescens* where only one individual was available and *P. c. concinna* where no sample was available. We also included two individuals from two outgroups – tree-kangaroos (*Dendrolagus – D. lumholtzi*) and pademelons (*Thylogale – T. thetis*), sister genera to *Petrogale* (see Potter et al. 2012a). Genomic DNA was extracted using a ‘salting out’ method (Sunnucks & Hales 1996).

*In-solution exon capture*

We identified target sequences for in-solution exon capture using a yellow-footed rock-wallaby (*P. xanthopus*) transcriptome (Bragg et al. 2016a). To do this, we began with a list of unique protein coding exons in the *Sarcophilus harrisii* (Tasmanian devil) genome (derived from the GTF file, Ensembl release 74, Flicek et al. 2014). We identified orthologs of these exonic sequences (truncated at intron boundaries) in the rock-wallaby transcriptome based on a reciprocal best BLAST (blastn) hit. We filtered this list of putative orthologs to a final set of 3960 target exons by removing short sequences (< 200 bases) and sequences that showed evidence of paralogy, based on BLAST hits to the *S. harrisii* or *Macropus eugenii* (tammar wallaby) genomes. Probes were then synthesized for these targets in a SeqCap EZ Developer Library (Roche NimbleGen). This capture targets a total of 1.83 Mb exon sequences. Scripts used to identify targets are available in Dryad Repository (on submission). Note, this approach has shown success of captured loci for taxa ~20 My diverged (Bragg et al. 2016b), and therefore is suitable at our timescales of divergence.

We made genomic libraries for each individual sample following the protocol of Meyer & Kircher (2010), using modifications of Bi et al. (2013). We added an additional modification, which included a double-bead clean up prior to the blunt-end repair step to remove fragments <200bp and >500bp. Each sample was given a unique barcode as part of this protocol and then libraries were quantified using a LabChip® DS Droplet Spectrophotometer (PerkinElmer) and pooled in equimolar amounts for hybridization. In each hybridization reaction, 56 individuals were pooled into one reaction totaling 1.2ug of genomic libraries, together with 5ug of mouse Cot-1 DNA (Life Technologies Corporation) and 56 barcode specific blocking oligos (1000 pmol) designed to block the unique barcodes and adapters used in the Meyer & Kircher (2010) protocol. We followed the manufacturer’s protocol (SeqCap EZ Developer Library) and hybridized the probes with the remaining ingredients for ~68-72 hours. These samples were run across a total of three separate hybridization experiments. After hybridization and clean up following the protocol, two independent enrichment PCRs of post-capture libraries were run (17 cycles). We did quality control checks after the experiments, performing a qPCR of pooled libraries pre- and post-capture using the DyNAmo Flash SYBR green qPCR kit (Thermo Fisher Scientific Inc.) to assess global enrichment efficiency (following Bi et al. 2012). Specifically, we amplified target loci from the capture, together with non-targets known to amplify but not in the capture (control) to confirm enrichment of targets and de-enrichment of non-targets. After confirmation of enrichment, we also ran post-capture libraries on a BioAnalyzer (2100; Agilent Technologies, Inc.) to check the quality and quantity. Once the post-capture libraries passed quality control checks we then proceeded with sequencing on a single lane of an Illumina HiSeq 2500 (100 bp paired-end run) at the ACRF Biomolecular Resource Facility.

*Bioinformatics*

Illumina sequencing reads were cleaned following a workflow developed by Singhal (2013). Briefly, this workflow removes duplicate, contaminant (human: GRCh37, ensemble version 67; *Escherichia coli*: str. K-12 substr. MG1655, GenBank: U00096.2) and low complexity reads. Then, TRIMMOMATIC (v 0.22; Bolger et al. 2014) is used to remove adaptors and low quality bases, and FLASH (v 1.2.2; Magoc & Salzber 2011) is used to merge overlapping read pairs. All scripts used in this analysis are archived in Dryad Repository (on submission).

The sequences for each locus in each sample were then assembled *de novo* from the cleaned sequencing reads, and heterozygous sites were identified and phased using a workflow described in Bragg et al. (2016a). Briefly, this this workflow began by identifying sequencing reads with homology to each of the target exons using BLAST (BLASTALL v 2.2.25, program = BLASTX; Altshul et al. 1990). The homologous sequencing reads were then assembled using VELVET (K values of 31, 41, 51, 61, and 71; v 1.2.08; Zerbino & Birney 2008). Contigs assembled using different K values were combined with CAP3 (Huang & Madan 1999), aligned to the targeted *Sarcophilus* protein, and trimmed to the boundaries of the target exon, using EXONERATE (v. 2.2.0; Slater & Birney 2005). If multiple contigs were identified, we found the one with the strongest BLASTX hit (maximum bit score) and ensured it did not have a stronger BLASTX hit to any other *Sarcophilus* protein. All scripts and procedures for assembling these sequences are archived in Dryad Repository (on submission).

Heterozygous sites for individuals were identified by mapping clean sequencing reads to the set of best assembled contigs using BOWTIE 2 (v 2.2.2; Langmead & Salzber 2012), and GATK (version 3.3-0-g37228af; McKenna et al. 2010) was used to identify and phase heterozygous sites (minimum genotype quality of GQ=20) in two hapltotype sequences for each locus in each sample.

Finally, haplotypes sequences obtained from the steps above were aligned and filtered using the EAPhy (v1.2; Blom 2015) pipeline. This uses MUSCLE (v 3.8.31; Edgar 2004) to align haplotype sequences, performs checks of coding frame to ensure amino acid coding (starting in frame 1), removes missing data from the ends of alignments and creates data files ready for analysis (fasta and phylip format) allowing for different amounts of missing data (we set 0-10%).

We identified our target sequences on the scaffolds of the *M. eugenii* genome assembly using BLAST (blastn). Based on knowledge of scaffold physical maps, and relationship of tammar wallaby chromosome to homologous *Petrogale* chromosomes (O’Neill et al. 1999; Eldridge & Close 1997), we were able to group loci into chromosomes (~400 loci were mapped to chromosomes). We then aligned multiple sets of concatenated sequences for analysis: X-chromosome, autosomal, rearranged autosomes focused on *penicillata* group rearrangements (5, 6, 9, 10), non-rearranged autosomes (2, 4, 7, 8). These alignments were created using the EAPhy pipeline described above and included loci with no missing individuals. In total, we had 21 loci for the X chromosome (8951 bp); 160 loci for the rearranged chromosomes (36,168 bp); and 140 loci for the non-rearranged chromosomes (75, 926 bp). Average pairwise divergences across the X, rearranged autosomal and non-rearranged autosomal loci were estimated for concatenated alignments using DnaSP (Librado and Rozas 2009). Averages were then estimated within each chromosomal group and divided by the total length of the alignment of the X, rearranged autosomal and non-rearranged autosomal loci to get comparisons for average net divergence.

*Phylogenetic Analysis*

1. *Mitogenome ­*– We assembled whole mitochondrial genomes from sequence capture by-catch, using a hybrid assembly – mapping approach. In short, we first used an iterative baiting and mapping strategy (MITObim; Hahn et al. 2013) to reconstruct mitochondrial genomes for four species, spread across the phylogeny, using the *Macropus robustus* (common wallaroo) mitochondrial genome as initial seed (Genbank Y10524.1). We then generated a rock-wallaby mitochondrial reference genome by aligning the four individuals using MUSCLE and calling a majority rule consensus sequence via visual inspection. The rock wallaby consensus sequence was then used as the initial seed for inferring mitochondrial genomes for each species using MITObim. We subsequently mapped the clean reads for each individual back against the inferred mitochondrial genome for that specific individual using Bowtie2, improved genotype calling (using the SAMtools workflow) and masked sites with sequencing coverage below 3x. Finally, we excluded each individual with more than 50% missing data and generated a mitochondrial genome alignment using MUSCLE.

We then aligned coding portions of the mitogenome in Geneious (v8.0.5; Kearse *et al.* 2012), including: ATP6, ATP8, ND1-ND6, COI-COII, cytb and concatenated these into a single alignment per individual (11,373 bp). We then estimated the mitochondrial phylogeny using a maximum likelihood approach in RAxML (Stamatakis 2014) using the rapid bootstrap algorithm. Analysis included, a random starting tree, 100 bootstrap replicates and used the GTR + Γ model.

1. *Concatenation* – we used a total evidence phylogeny based on all of the concatenated nuclear loci, as current bioinformatics treatment restricts loci to just the exons (conservative alignment at phylogenetic scale). Because individual gene trees at this level are poorly resolved we could not apply discordance analyses (e.g. BUCKy), hence the focus here is on concatenation across loci with common properties (see below). We estimated the phylogenetic relationships of both ambiguous data and phased haplotypes using a concatenated alignment and maximum likelihood approach in RAxML (only phased haplotypes shown). We implemented the rapid bootstrap algorithm, starting from a random starting tree, with 100 bootstraps using the GTR + Γ model. Analysis was performed using a 1+2 and 3 codon partition model, and another with no partition. Given the concordance across runs, we only include the no partition haplotype results. The consensus tree and bootstrap support were estimated for the combined dataset (all loci), the X-chromosome loci only, the autosomal loci only, the rearranged loci (5, 6, 9, 10) and the non-rearranged loci (2, 4, 7, 8).

*(iii) Mapping ancestral chromosomal states on phylogeny*

Ancestral chromosomal states were estimated across the *Petrogale* phylogeny using ancestral state reconstruction in Mesquite (Maddison & Maddison 2016). The concatenated phylogeny topology was used as the species tree (using just a single lineage to represent chromosomally different taxa) and a chromosomal matrix of character states was created to account for all chromosomal rearrangements (i.e. inversions, fusions, centric shifts; see Supp. Table 2 for character matrix). Shared character states included taxa with both centric shifts and fusions, as we were uncertain as to the order of these rearrangements. Reconstruction was performed using the maximum parsimony approach and analyzed chromosome by chromosome. This reconstructs the ancestral history that minimizes the total number of character-state changes. We explore the development of independent chromosomal rearrangements evolving across the *Petrogale* phylogeny.

*(iv)Phylogenetic Network Approaches* – we estimated phylogenetic networks using two approaches, an exploratory statistical approach (Splits tree – Huson & Bryant 2006) and a model based approach (PhyloNet – Than et al. 2008), where the first approach suggested reticulation. We ran Neighbor-Net (Bryant & Moulton 2002) in Splits tree, which clusters individuals using a distance matrix based on average distances to construct a phylogenetic network. This was run on the entire concatenated nuclear dataset for the *brachyotis*, *lateralis* and *penicillata* groups. We then evaluated reticulation using the maximum likelihood approach to infer a species network given a specified number of reticulations using the program PhyloNet (Yu et al. 2013, 2014). Given the computational limits of the approach (combine # loci, # individuals, # reticulation events), we used a subset of the data to explore evidence of reticulations (introgression and incomplete lineage sorting) in the evolutionary history of *Petrogale*. In particular, we were interested in comparing three groups: the *brachyotis* group, the *lateralis* group and the *penicillata* group (*P. assimilis*, *P. mareeba*, *P. sharmani*). We subset the alignments into these chromosomal groups (*penicillata* group – three species) and used *Dendrolagus* as an outgroup. Two separate analyses were performed on the h0 for one of the two individuals for each species to compare phylogenetic network results. For PhyloNet, alignments of these subsets of individuals were created using EAPhy. Then, gene trees were estimated for each taxon group using maximum likelihood analysis in RAxML using the same methods outlined above (Dryad Repository – on submission). The best tree from each gene tree analysis was combined for PhyloNet analysis. We then estimated the phylogenetic network using 0, 1, 2 and 3 reticulation nodes, using default parameters, analyzing 50 runs and saving the top 5 networks. The best network (lowest log likelihood) was used in log likelihood comparisons of the various models of reticulation. To evaluate the best fitting model of reticulation to our network, we compared log likelihood scores (lnL) and Akaike Information Criterion (AIC), AIC = 2k - 2lnL, where 2k = number of free parameters. The lowest AIC value was used as the best fitting model of reticulation given the data.

**References – Materials & Methods**

Asthul, S.F., Gish, W., Miller, W., Myers, E.W., Lipman, D.J. (1990). Basic local alignment search tool. *J. Mol. Biol.* 215, 403-401.

Bi, K., Vanderpool, D., Singhal, S., Linderoth, T., Moritz, C., Good, J.M. (2012). Transcriptome-based exon capture enables highly cost-effective comparative genomic data collection at moderate evolutionary scales. *BMC Genomics* 13, 403.

Bi, K., Linderoth, T., Vanderpool, D., Good, J.M., Nielsen, R., Moritz, C. (2013). Unlocking the vault: nextgeneration museum population genomics. *Mol. Ecol.* 24, 6018-6032.

Blom, M. (2015). EAPhy: a flexible tool for high-throughput quality filtering of exon-alignments and data processing for phylogenetic methods. *PLoS currents* 7

Bolger, A.M., Lohse, M., Usadel, B. (2014). Trimmomatic: a flexible trimmer for Illumina sequence data. *Bioinformatics* 30, 2114-2120.

Bragg, J.G., Potter, S., Bi, K., Catullo, R., Donnellan, S.C., Eldridge, M.D., Joseph, L., Keogh, J.S., Oliver, P., Rowe, K.C., Moritz, C., (2016a). Resources for phylogenomic analyses of Australian terrestrial vertebrates. *Mol. Ecol. Res.* doi:10.1111/1755-0998.12633

Bragg, J.G., Potter, S., Bi, K., Moritz, C. (2016b). Exon capture phylogenomics: efficacy across scales of divergence. *Mol. Ecol. Res.* 16, 1059-1068.

Bryant, D., and Moulton, V. (2002). “NeighborNet: an agglomerative method for the construction of planar phylogenetic networks,” in International Workshop on Algorithms in Bioinformatics, (Springer Berlin Heidelberg), 375-391.

Edgar, R.C. (2004). MUSCLE: multiple sequence alignment with high accuracy and high throughput. *Nucleic Acids Res.* 32, 1792-1797.

Eldridge, M.D.B., and Close, R.L. (1997). Chromosomes and evolution in rock-wallabies, *Petrogale* (Marsupialia: Macropodidae). *Aust. Mammal.* 19, 123-136.

Flicek, P., Arnode, M.R., Barrell, D., Beal, K., Billis, K., Brent, S., Carvalho-Silva, D., Clapham, P., Coates, G., Fitzgerald, S., Gil, L., Girón, C.G., Gordon, L., Hourlier, T., Hunt, S., Johnson, N., Juettemann, T., Kähäri, A.K., Keenan, S., Kulesha, E., Martin, F.J., Maurel, T., McLaren, W.M., Murphy, D.N., Nag, R., Overduin, B., Pignatelli, M., Pritchard, B., Pritchard, E., Riat, H.S., Ruffier, M., Sheppard, D., Taylor, K., Thormann, A., Trevanion, S.J., Vullo, A., Wilder, S.P., Wilson, M., Zadissa, A., Aken, B.L., Birney, E., Cunningham, F., Harrow, J., Herrero, J., Hubbard, T.J., Kinsella, R., Muffato, M., Parker, A., Spudich, G., Yates, A., Zerbino, D.R., Searle, S.M. (2014). Ensembl 2014. *Nuc. Acids Res.* 42, Database issue: D749-D755.

Hahn, C., Bachmann, L., Chevreux, B. (2013). Reconstructing mitochondrial genomes directly from genomic next-generation sequencing reads - a baiting and iterative mapping approach. *Nucleic Acids Res.* 41: e129. doi: 10.1093/nar.gkt371

Huang, X., and Madan, A. (1999). CAP3: A DNA sequence assembly program. *Genome Res.* 9, 868-877.

Huson, D.H., and Bryant, D. (2006). Application of phylogenetic networks in evolutionary studies. *Mol. Biol. Evol*. 23, 254-267.

Kearse, M., Moir, R., Wilson, A., Stones-Havas, S., Cheung, M., Sturrock, S., Buxton, S., Cooper, A., Markowitz, S., Duran, C., Thierer, T., Ashton, B., Mentjies, P., Drummond, A. (2012). Geneious Basic: an integrated and extendable desktop software platform for the organization and analysis of sequence data. B*ioinformatics* 28, 1647-1649.

Langmead, B., and Salzberg, S. (2012). Fast gapped-read alignment with Bowtie 2. *Nature Methods* 9, 357-359.

Librado, P., and Rozas, J. (2009). DnaSP v5: A software for comprehensive analysis of DNA polymorphism data. Bioinformatics 25: 1451-1452.

Maddison, W.P., and Maddison, D.R. (2016). Mesquite: a modular system for evolutionary analysis. Version 3.10 http://mesquiteproject.org

Magoc, T., and Salzberg, S. (2011). FLASH: fast length adjustment of short reads to improve genome assemblies. *Bioinformatics* 27, 2957-63.

McKenna, A.H., Hanna, M., Banks, E., Sivachenko, A., Cibulskis, K., Kernytsky, A., Garimella, K., Altshuler, D., Gabriel, S., Daly, M., DePristo, M.A. (2010). The genome analysis toolkit: a MapReduce framework for analyzing next-generation DNA sequencing data. *Genome Res.* 20, 1297-1303.

Meyer, M., and Kircher, M. (2010). Illumina sequencing library preparation for highly multiplexed target capture and sequencing. *Cold Spring Harbor Protocol*, pdb.prot5448. doi:10.1101/pdb.prot5448

O'Neill, R.W., Eldridge, M.D.B., Toder, R., Ferguson-Smith, M.A., O'Brien, P.C., Graves, J.A.M. (1999). Chromosome evolution in kangaroos (Marsupialia: Macropodidae): cross species chromosome painting between the tammar wallaby and rock wallaby spp. with the 2 n= 22 ancestral macropodid karyotype. *Genome* 42, 525-530.

Potter, S., Cooper, S.J., Metcalfe, C.J., Taggart, D.A., Eldridge, M.D.B. (2012a). Phylogenetic relationships of rock-wallabies *Petrogale* (Marsupialia: Macropodidae) and their biogeographic history within Australia. *Mol. Phylogenet. Evol*. 62, 640-652.

Singhal, S. (2013) De novo transcriptomic analyses for non-model organisms: an evaluation of methods across a multi-species data set. *Mol. Ecol. Res.* 13, 403-416.

Slater, G.S.C., and Birney, E. (2005). Automated generation of heuristics for biological sequence comparison. *BMC Bioinformatics* 6, 31.

Stamatakis, A. (2014). RAxML version 8: a tool for phylogenetic analysis and post-analysis of large phylogenies. *Bioinformatics* 30, 1312-1313.

Suh, A. (2016). The phylogenomic forest of bird trees contains a hard polytomy at the root of Neoaves. *Zool. Scripta* 45, 50-62.

Sunnucks, P. and Hales, D.F. (1996). Numerous transposed sequences of mitochondrial cytochrome oxidase I-II in aphids of the genus *Sitobion* (Hemiptera: Aphididae). *Mol. Biol. Evol.* 13, 510-524.

Than, C., Ruths, D., Nakhleh, L. (2008). PhyloNet: a software package for analyzing and reconstructing reticulate evolutionary histories. *BMC Bioinformatics* 9, 322.

Yu, Y., Ristic, N., Nakhleh, L. (2013). Fast algorithms and heuristics for phylogenomics under hybridization and incomplete lineage sorting. *BMC Bioinformatics* 14, S6.

Yu, Y., Dong, J., Liu, K., Nakhleh, L. (2014). Maximum likelihood inference of reticulate evolutionary histories. *Proc. Natl. Acad. Sci. U.S.A.* 111, 16448-16453.

Zerbino, D.R., and Birney, E. (2008). Velvet: algorithms for de novo short read assembly using de Bruijn graphs. *Genome Res.* 18, 821-829.

Zickler, D., and Kleckner, N. (1999). Meiotic chromosomes: integrating structure and function. *Annu. Rev. Genet*. 33, 603-754.
